# Apolipoprotein E allele 4 effects on Single-Subject Gray Matter Networks in Mild Cognitive Impairment

**DOI:** 10.1016/j.nicl.2021.102799

**Published:** 2021-08-24

**Authors:** Gretel Sanabria-Diaz, Jean-Francois Demonet, Borja Rodriguez-Herreros, Bogdan Draganski, Ferath Kherif, Lester Melie-Garcia

**Affiliations:** a LREN, Department of Clinical Neurosciences, Lausanne University Hospital (CHUV), Rue du Bugnon 46, Mont Paisible 16, Lausanne, Vaud 1011, Switzerland; b Faculty of Biology and Medicine, University of Lausanne (UNIL), Rue du Bugnon 21, Lausanne CH-1011, Switzerland; c Leenaards Memory Center, Lausanne University Hospital (CHUV), Rue du Bugnon 21, Mont Paisible 16, Lausanne 1011, Switzerland; d Cantonal Autism Center, University of Lausanne. University Hospital Lausanne. Av. de Beaumont 23, Lausanne 1011, Switzerland; e Max-Planck-Institute for Human Cognitive and Brain Sciences, Postfach 500355, Leipzig D-04303, Germany; f Applied Signal Processing Group (ASPG), Swiss Federal Institute of Technology Lausanne (EPFL), Route Cantonale, Lausanne 1015, Switzerland; g Translational Imaging in Neurology Group (ThINk), Department of Biomedical Engineering, University of Basel and University Hospital Basel, Gewerbestrasse 14, Allschwil., Basel 4123, Switzerland

**Keywords:** Mild Cognitive Impairment, Alzheimer Disease, Gray matter networks, Single-Subject Gray Matter Networks, Graph theory

## Abstract

There is evidence that gray matter networks are disrupted in Mild Cognitive Impairment (MCI) and associated with cognitive impairment and faster disease progression. However, it remains unknown how these alterations are related to the presence of Apolipoprotein E isoform E4 (ApoE4), the most prominent genetic risk factor for late-onset Alzheimer’s disease (AD). To investigate this topic at the individual level, we explore the impact of ApoE4 and the disease progression on the Single-Subject Gray Matter Networks (*SSGMNets*) using the graph theory approach. Our data sample comprised 200 MCI patients selected from the ADNI database, classified as non-Converters and Converters (will progress into AD). Each group included 50 ApoE4*-*positive (‘Carriers', ApoE4 + ) and 50 ApoE4*-*negative ('non-Carriers', ApoE4-). The *SSGMNets* were estimated from structural MRIs at two-time points: baseline and conversion. We investigated whether altered network topological measures at baseline and their rate of change (RoC) between baseline and conversion time points were associated with ApoE4 and disease progression. We also explored the correlation of *SSGMNets* attributes with general cognition score (MMSE), memory (ADNI-MEM), and CSF-derived biomarkers of AD (Aβ42, T-tau, and P-tau). Our results showed that ApoE4 and the disease progression modulated the global topological network properties independently but not in their RoC. MCI converters showed a lower clustering index in several regions associated with neurodegeneration in AD. The *SSGMNets'* topological organization was revealed to be able to predict cognitive and memory measures. The findings presented here suggest that *SSGMNets* could indeed be used to identify MCI ApoE4 Carriers with a high risk for AD progression.

## Highlights

• ApoE4-related changes on gray matter networks are associated with AD progression in MCI.
• Gray matter topological organization predicts a steeper decline in cognitive functioning.
• Network topological measures might detect progression to dementia in MCI patients.

## 1 Introduction

Late-Onset Alzheimer’s Disease (LOAD) is the most common cause of dementia, accounting for 60% to 80% of cases (Hardy, 1997). However, there are currently no disease-modifying treatments and clinical drug trials have a high failure rate (Cummings et al., 2014). The postulate for this result is that AD brain pathology begins years before the cognitive decline. Consequently, in recent years, research has moved toward the study of the earliest clinical signs of neurodegeneration that are likely to evolve to AD. In this effort, a particular interest has been dedicated to Mild Cognitive Impairment (MCI) as a transitional phase between the cognitive changes associated with aging and early AD (Petersen, 2004, Petersen et al., 2001). It is a window in which it may be possible to intervene and modulate the disease progression (Albert et al., 2011, Gauthier et al., 2006, Mueller et al., 2005, Petersen et al., 1999).

However, MCI is especially challenging because of the considerable variability in terms of individual clinical outcomes (Jack et al., 2013, Scheltens, 2013), dependent on multiple genetic and environmental risk factors involved in AD pathogenesis (Collie and Maruff, 2000, Petersen, 2000). In terms of genetic markers, the best-established genetic risk factor for AD is the Apolipoprotein E (ApoE) ε4 allele (ApoE4) (for reviews, Bekris et al., 2010, Bookheimer and Burggren, 2009; Liu et al., 2013).

While there is evidence linking ApoE4 to cognitive deficits, morphological, structural, and functional brain alterations during AD progression (Cherbuin et al., 2007; Liu et al., 2013), at this point, it is still unclear how this genetic risk factor affects the organization of brain networks.

One viable mathematical approach to elucidate the ApoE4 impact on MCI brain networks is graph formalism (Boccaletti et al., 2006). In graph theory, our brain is studied as a model composed of some basic elements -nodes- (brain regions) and their relationship (edges); and the brain’s complex co-variance patterns are translated into global and regional graph metrics (Bullmore and Bassett, 2011). During the past decade, graph analysis has already been applied to study the structural co-variance networks in AD and MCI (Alexander-Bloch et al., 2013). It is based on the phenomenon that regions that are correlated in morphometric descriptors (e.g., cortical thickness) are highly probable to be part of the same brain system underlying particular behavioral and cognitive functions (Lerch et al., 2006).

Using graph theory formalism, previous studies found evidence of the ApoE4-related modulation on healthy aging, MCI, and the AD co-variance brain network based on physiological variables derived from different image modalities (rsFMRI, FDG-PET, and DWI) (Brown et al., 2011, Giau et al., 2015, Goryawala et al., 2015, Li et al., 2019, Sanabria-Diaz et al., 2021, Seo et al., 2013, Wang et al., 2015, Yao et al., 2015, Zhu et al., 2018). Their findings propose a link between the possession of ApoE4 and brain network organization abnormalities in AD, suggesting disease-related disconnection mechanisms (Delbeuck et al., 2003, Filippi and Agosta, 2011, He et al., 2009). Moreover, these studies have provided new insights into the understanding of the biological mechanism of AD. They could lead to the use of a network-based imaging biomarker for MCI diagnosis and monitoring.

Nevertheless, it is essential to highlight that the inclusion of MCI is only reported by one previous study conducted by Yao et al. (Yao et al., 2015). Yet, in this case, the sample design makes the interpretation of the MCI brain network topology challenging as the groups (NC, MCI, and AD) were pulled together to conform the Carriers and non-Carriers samples. Additionally, only one study used data from structural Magnetic Resonance Imaging (sMRI) to construct the brain network structural co-variance, in this case, applied to a healthy aging sample (Goryawala et al., 2015). sMRI is an attractive technique because of the wide availability in clinical and research settings and its high anatomical resolution compared with other neuroimaging modalities (i.e PET).

Recently our group published the first study about the ApoE4-related effects on the structural co-variance brain network topology in MCI (Sanabria-Diaz et al., 2021). We found that the E4 allele shaped the topological organization of cortical thickness networks in this phase. Our results revealed several network measures alterations in MCI Carriers (ApoE4-positive) compared to non-Carriers, such as a decrease in global and homologous connectivity strength, clustering index, characteristic path length, local efficiency, modularity, and an increase of global efficiency. In general, they support an aberrant network topology associated with the genetic risk, which was not detectable with a standards univariate approach.

However, this study has limitations. First, we applied a group-based graph analysis that generated one correlation matrix per group. This method did not allow us to establish associations between the network properties changes and other AD biomarkers (i.e., CSF Aβ42, tau, hippocampal atrophy) at the individual level. In our opinion, it is a critical aspect to gain insight into the biological meaning of the structural co-variance network alterations. Second, the study was based on a cross-sectional design where information about the final clinical outcome was not included. As MCI is highly heterogenic in terms of prognosis, the progression into AD is essential to control confounding effects and relate the ApoE4 to the brain network topological alterations. Finally, ApoE4 and AD progression's interaction effects were not analyzed, making it challenging to disentangle each factor's implication on the results.

In the recent past, the group-based graph analysis limitation has been overcome by a methodology that constructs single-subject brain networks from the native gray matter segmentation space (Dicks et al., 2020, Dicks et al., 2018; Tijms et al., 2012, Tijms et al., 2013a, Tijms et al., 2014, Tijms et al., 2018). This method accounts for the similarity in gray matter structure between brain areas measured with sMRI (Mechelli et al., 2005, Tijms et al., 2012). These co-variance patterns have been associated with coordinated growth trajectories of gray matter during development, functional co-activation, and axonal connectivity (Alexander-Bloch et al., 2013, Gong et al., 2012).

Few prior studies applied this approach to study *Single-Subject Gray Matter Networks* (*SSGMNets*) in AD and MCI (Dicks et al., 2020, Dicks et al., 2018; Tijms et al., 2013a, Tijms et al., 2016, Tijms et al., 2018). These studies show that the more network topology randomness, the worse cognitive impairment level in AD patients (Tijms et al., 2013a, Tijms et al., 2014). Additionally, in preclinical phases, the network measures changes predicted hippocampal atrophy rate and faster atrophy in other areas associated with AD progression (Dicks et al., 2020). In MCI, the network measures showed sensitivity to initial structural alterations related to amyloid deposition (Tijms et al., 2018). The network properties alteration revealed a relation with cognitive impairment and a faster decline in almost all cognitive domains (Dicks et al., 2018).

Despite these preceding findings, we are unaware of any studies that focused on how ApoE4 affects *SSGMNets* in MCI. Moreover, no neuroimaging study has so far explored the interaction between ApoE4 and disease progression on these networks' topological properties in MCI. Such longitudinal follow-ups are required to reveal changes associated with the genetic risk allele per se and to be able to identify possible network properties alterations related to subsequent progression into AD. Additionally, the ApoE4 modulation on the association between the network topology and other neuropathological AD biomarkers (e.g., CSF amyloid β 42 (Aβ42) and total tau levels) in MCI is still unexplored.

This paper precisely addresses these crucial questions and provides experimental evidence of MCI pathological processes, which may help implement future strategies to prevent or delay the progression into AD.

Importantly, we focused our study on MCI without and with one ε4 allele that are the most representative subgroups present in AD. A body of evidence indicates that the combination of the ε3-ε3 alleles is the most frequent in cognitively healthy individuals with a frequency of 62.3 %, followed by ε3-ε4 = 22.2 %, and ε4-ε4 with only 1.9 %. However, in AD, ε3-ε4 shows the highest frequency with 43.4 %, followed by the ε3-ε3 with 34.3 % (“AlzGen, n.d.) (ALZGENE 2010, http://www.alzgene.org/meta.asp?geneID=83).

## 2 Materials and methods

### 2.1 Participants

Data used in the preparation of this article were obtained from the Alzheimer's Disease Neuroimaging Initiative (ADNI) database (http://adni.loni.usc.edu). The ADNI was launched in 2003 as a public-private partnership, led by Principal Investigator Michael W. Weiner, MD. The primary goal of ADNI has been to test whether serial magnetic resonance imaging (MRI), positron emission tomography (PET), other biological markers, and clinical and neuropsychological assessment can be combined to measure the progression of mild cognitive impairment and early Alzheimer's disease. ADNI was approved by the institutional review board of all participating institutions, and written informed consent was obtained from all participants at each site. For up-to-date information, see www.adni-info.org. Full details of subject recruitment, biomarkers as quantitative phenotypes, MRI scanning protocols, and data pre-processing were published elsewhere (Jack et al., 2010b; Mueller et al., 2005, Saykin et al., 2010) (http://www.loni.ucla.edu/ADNI/), and only a brief account is given here.

We selected participants with a clinical diagnosis of MCI who had completed at least two visits and fulfilled ADNI inclusion/exclusion criteria (http://adni.loni.usc.edu/wp-content/uploads/2010/09/ADNI_GeneralProceduresManual.pdf.). Details of clinical diagnostic criteria have been previously described in (Aisen et al., 2015, Petersen et al., 2010). Briefly, the inclusion criteria were as follows: Mini-Mental-State-Examination (MMSE) scores between 24 and 30 (inclusive), a memory complaint, objective memory loss measured by education adjusted scores on the Wechsler Memory Scale Logical Memory II (Aisen et al., 2015, Petersen et al., 2010), a Clinical Dementia Rating (CDR) of 0.5, and absence of significant levels of impairment in other cognitive domains, essentially preserved activities of daily living and a lack of dementia.

Exclusion criteria included: 1) the presence of a major depressive disorder or significant symptoms of depression; 2) modified Hachinski ischemia score greater than five; 3) significant neurological or psychiatric illness; 4) use of antidepressant drugs with anticholinergic side effects; 5) high dose of neuroleptics, chronic sedatives, hypnotics, antiparkinsonian medication, and use of narcotic analgesics. Details about inclusion/exclusion criteria can be found in http://adni.loni.usc.edu/wp-content/themes/freshnewa-dev-v2/clinical/ADNI-1_Protocol.pdf) (Petersen et al., 2010).

We stratified the MCI group into those with only one ApoE4 allele (ApoE4+, Carriers) and those without (ApoE4-, non-Carriers).

We excluded participants with the E2 allele due to the possible protective effects (Serrano-Pozo et al., 2015). ApoE genotyping was performed at the time of participant enrollment and included in the ADNI database. The samples were sent to the ADNI Biomarker Core at the University of Pennsylvania within 24 h of collection for analysis. ApoE genotyping details can be accessed at http://adni.loni.usc.edu/data-samples/clinical-data/(Saykin et al., 2010). For genotyping methods see www.ADNI.org.

After controlling all the inclusion/exclusion parameters and the existence of the ApoE4 allele in the ADNI database, we selected a sample of 200 late MCI participants subdivided into 100 late MCI non-converters and 100 converters to AD (Table 1). Each subgroup was subdivided into 50 Carriers (ApoE4 + ) and 50 non-Carriers' (ApoE4-) patients. More information about the procedure for groups selection can be found in Supplementary Material.

**Table 1:** Demographics and Clinical Characteristics of the MCI non-converters and Converters. MMSE, Gray matter Volume, normalized Gray matter volume, CDR were found differences between groups (in bold), specifically in MCI that converted to AD in the second diagnosis time.

	MCI non-Converters				MCI Converters			
	Diagnosis Time 1 (MCI)		Diagnosis Time 2 (MCI)		Diagnosis Time 1 (MCI)		Diagnosis Time 2 (AD)	
	Carriers	non-Carriers	Carriers	non-Carriers	Carriers	non-Carriers	Carriers	non-Carriers
# of Participant	50	50	50	50	50	50	50	50
males/females	34/16	36/14	34/16	36/14	37/13	36/14	37/13	36/14
Age, years	74.76 (7.12)	76.33 (7.85)	77.06 (7.04)	77.71 (7.92)	74.18 (6.74)	75.71 (8.52)	77.47 (7.12)	78.71 (8.84)
Education, years	15.76 (2.99)	15.68 (2.86)	15.76 (2.99)	15.68 (2.86)	16.66 (2.49)	16.50 (2.57)	16.66 (2.49)	16.50 (2.57)

**Average MMSE**	**27.46 (1.85)**	**27.76 (1.67)**	**27.40 (1.68)**	**27.80 (1.85)**	**27.10 (1.46)**	**26.60 (1.69)**	**21.34 (3.71)**	**23.06 (3.87)**
**CDR 0.5**	**50**	**50**	**50**	**50**	**50**	**50**	**15**	**20**
**CDR 1**	na	na	na	na	na	na	**28**	**23**
**CDR 2**	na	na	na	na	na	na	**7**	**7**
TIV	1592.3 (167.2)	1591.6 (174.1)	1569.8 (202.9)	1584.6 (198.9)	1602.2 (192.7)	1591.7 (177.5)	1602.3 (192.5)	1577.2 (196.7)

**Gray matter volume**	**608.5 (63.8)**	**598.2 (70.11)**	**585.3 (79.98)**	**585.2 (85.38)**	**593.6 (82.25)**	**575.9 (58.97)**	**561.6 (81.57)**	**541.9 (86.71)**
**Normalized Gray matter volume**	**0.40 (0.04)**	**0.39 (0.04)**	**0.39 (0.04)**	**0.38 (0.05)**	**0.39 (0.04)**	**0.38 (0.03)**	**0.38 (0.04)**	**0.36 (0.05)**
Network size	7486.68 (651.8)	7420.5 (649.10)	7339.72 (855.47)	7392.02 (664.41)	7420.86 (792.77)	7404.6 (681.54)	7287.72 (810.93)	7226.92 (918.19)

Data are presented as number or mean and standard deviations (SD). MMSE is Mini-mental state examination, mm3: cubic millimeter, MCI is Mild Cognitive Impairment, CDR is Clinical Dementia Rate, TIV is total intracranial volume, na is not applicable.

### 2.2 Cognitive and biomarker measures

We used a composite score for memory (ADNI-MEM) using data from the ADNI neuropsychological battery. The ADNI‐MEM description can be found in (Crane et al., 2012). The authors described the composite score as an empirically derived memory measure that includes items from four memory tests available within the ADNI test battery, including the ADAS‐Cog, the Rey Auditory Verbal Learning Test (RAVLT) (Rey, 1958), Logical Memory from the Wechsler Memory Scale (Wechsler, 1945), and the word list from the Mini‐Mental State Examination (Folstein et al., 1975). ADNI-MEM has been validated in published papers (Folstein et al., 1975; Jack et al., 2010a; Jack and Holtzman, 2013). The ADNI-MEM scores for each ADNI participant at each study visit are reported in the UWNPSYCHSUM file. More details about the cognitive tests can be found at ADNI website (http://adni.loni.usc.edu/methods/documents/) under clinical protocols. The individual cognitive measurements downloaded from the ADNI website (https://ida.loni.usc.edu/pages/access/studyData.jsp?categoryId = 12&subCategoryId = 36) and the composite ADNI-MEM method information can be found at (UW – Neuropsych Summary Scores Methods.pdf).

### 2.3 Biomarker measurements in CSF: Aβ42, P-tau, T-tau.

In the present study, we used the CSF core biomarkers measurements for AD performed with the Elecsys® total-tau CSF, the Elecsys® Phospho-Tau (181P) CSF, and the Elecsys® β-amyloid (1–42) CSF immunoassays on a Cobas E 601 instruments (Hansson O et al., 2018; Bittner et al., 2016). The data is available in the 'UPENNBIOMK9.csv' file at the ADNI database (downloaded on May 11th, 2019). The analyzed measuring ranges of these assays are the following: 80 to 1300 pg/ml for total-Tau CSF, 8 to 120 pg/ml for Phospho-Tau (181P) CSF, and 200 to 1700 pg/ml for Elecsys® β-Amyloid (1–42) CSF immunoassays. The CSF biomarkers have been shown to predict cognitive decline and progression to dementia in patients with MCI (Hansson et al., 2018, Shaw et al., 2009).

Details about CSF biomarker group classification based on the A/T/N scheme (Jack et al., 2016) are described in Supplementary Table S3.

### 2.4 MRI acquisition and pre-processing

Pre-processed versions of the 400 T1-weighted MRI scans were downloaded from LONI Image Data Archive. Further details are available in the ADNI-MRI technical procedures manual (ADNI-MRICore, 2005). Further details are available in the ADNI-MRI technical procedures manual (http://adni.loni.usc.edu/methods/documents/MRI protocols). Pre-processing steps can be found elsewhere (Jack et al., 2008). Images were pre-processed using Statistical Parametric Mapping software version 12 (SPM12) (https://www.fil.ion.ucl.ac.uk/spm/software/spm12/). First, the structural T1 weighted images are segmented into gray matter, white matter, and cerebrospinal fluid tissue classes using default settings. Next, 114  gray matter regions were parcellated based on Neuromorphometrics atlas using the Neuromorphometrics toolbox (Full list of structures listed in Supplementary Materials Table S1) (http://www.Neuromorphometrics.com/) to obtain individual anatomical atlases. This atlas has proved to show high sensitivity detecting age modulation on the networks of myelin co-variance topological features (Melie‐Garcia et al., 2018). The Total intracranial volume (TIV) was computed as the sum of gray and white matter and cerebrospinal fluid volumes in cm^3^. Normalized gray matter volume is defined as the ratio between gray matter volume and TIV.

The native T1-weighted images were re-oriented to the canonical MNI space of the SPM12 'avg305T1′ template using a rigid-body transformation and resliced to a voxel size 2×2×2 mm. Both transformations were applied to the native space gray matter segmented images and individual atlases using trilinear and nearest-neighbor interpolations, respectively. These pre-preprocessing steps help to standardize voxel sizes and reduce dimensionality.

We selected the CSF biomarkers and cognitive variables measurements with a maximum of two months to MRI study dates.

### 2.5 Single subject gray matter networks and its topological properties

#### 2.5.1 Extraction of single subject gray matter networks

Single Subject Gray Matter Networks (*SSGMNets*) were extracted from transformed gray matter segmentation using a method developed and published by Tijms et al. 2012 (https://github.com/bettytijms/Single_Subject_Gray_Matter_Networks; (Tijms et al., 2012)). This toolbox was implemented in MATLAB programming language (http://www.mathworks.com).

Briefly, to extract *SSGMNets*, each individual's gray matter segmentation is parcellated into multiple small cubes of 3×3×3 = 27 voxels each. These non-overlapping cubes serve as the 'nodes' in the network, thereby using geometrical information and gray matter density values (i.e., from the tissue segmentation) in the voxels. Their 'connection' refers to 'edges' indicating statistically similar gray matter morphology of two cubes as determined by calculating the Pearson's correlation. Notably, the term 'connection' in this methodology should not be confused with anatomical connections (axonal connections). The cortex is a curved object, and hence two similar cubes could be at an angle to each other, incorrectly decreasing similarity values (Tijms et al., 2012). Therefore, each seed node was rotated by an angle θ with multiples of 45 degrees and reflected over all axes to identify the target node's maximal similarity value. Nodes with zero variance in their gray matter density values were excluded (average across all subjects <0.01%) since, in this case, the correlation coefficient is undefined (Tijms et al., 2012).

All pairwise correlations are entries in a matrix denominated 'connectivity' or 'adjacency matrix' in graph theory terms. The presence or absence of connections between nodes is determined according to an individualized threshold with a random permutation method that ensures a maximum of 5% spurious connections for each *SSGMNets* (Tijms et al., 2012).

A correction for multiple comparisons was applied using the False Discovery Rate (FDR) to determine a corrected-threshold (FDR-threshold) (Benjamini and Hochberg, 1995, Benjamini and Yekutieli, 2001). The FDR-threshold was applied to binarize the *SSGMNet.* An edge (element of the connectivity matrix) indicated by '1′ occurs when a correlation is higher than the FDR-threshold. On the contrary, the absence of an edge is represented by 0 while the correlation is lower than FDR-threshold.

#### 2.5.2 Network properties computation: Graph theory approach

Formally, a complex network can be represented as a graph G = [N, K], the components of this system are called nodes (N), and K refers to the relations or connections between them are called edges (Boccaletti et al., 2006). In our case, the nodes are the cubes defined over the individual's gray matter segmentation, and the edges are derived from the statistical similarity in gray matter morphology between pairs of cubes (i.e., nodes).

Technically, we used the following global network attributes to characterize the *SSGMNet* topological organization. The attributes include the clustering coefficient (Clux) (i.e., the level of interconnectedness between the neighbors of a node), the characteristic path length (CharPath) (i.e., the minimum number of edges between any pair of nodes), the normalized clustering index (Clux-Normalized), the normalized characteristic path length (CharPath-Normalized), and global efficiency (Eglobal) (i.e., how efficiently the information can be exchanged over the network). The global connectivity (GConnect) is defined as the mean correlation of all the connectivity matrix elements.

To estimate Clux-Normalized (gamma) and CharPath-Normalized (lambda), we first constructed 20 randomized reference networks matching the original ones in size and degree distribution. The Clux and CharPath mean over the 20 random networks are calculated and denoted as Clux_rand and CharPath_rand. So, Clux-Normalized and CharPath-Normalized are defined as the ratios: Clux-Normalized = Clux/Clux_rand and CharPath-Normalized = CharPath/CharPath_rand (Humphries et al., 2008, Maslov and Sneppen, 2002, Watts and Strogatz, 1998). The small world (sigma) attribute is computed as the ratio between Clux-Normalized and CharPath-Normalized.

We estimated the normalized clustering index attribute for each node (Nodal_Clux-Normalized) to describe the network's nodal properties. Nodal_Clux-Normalized is defined as: Nodal_Clux-Normalized = Nodal_Clux/Clux_rand. Then, this measure is averaged across all nodes (cubes) for each of 114 anatomical structures defined in the Neuromorphometric atlas as the second step.

We define the sparsity of the networks as the density of the connection within the connectivity matrix. This measure was calculated as the percentage number of existing edges respect to the maximum number of possible edges (N × (N – 1), where N is the number of nodes).

All network measures were computed with functions from the Brain Connectivity Toolbox (www.brain-connectivity-toolbox.net). More information about the graph network topological properties definition and meaning can be found elsewhere (Rubinov and Sporns, 2010).

### 2.6 Rate of change analysis

We also were interested in evaluating the impact of ApoE4 and progression factors on the Rate of Change (RoC) of the *SSGMNets* topological properties, morphometric, psychological, and CSF variables. RoC provides information about how fast the variables change linearly with time. If a variable Y is defined in two-time points t1, t2 as Y(t1) and Y(t2), RoC is estimated as follows: RoC = ΔY/Δt where Δt = t2 – t1 and ΔY = Y(t2)-Y(t1). Also, RoC normalizes the variable's change between two-time points by the elapsed time. This time normalization step is necessary when subjects have different Δt between baseline and second visit time. The two-time points RoC estimation provides a local RoC surrogate measure between t1 and t2.

### 2.7 Statistical analysis

We checked the normal distributions of all variables using Kolmogorov–Smirnov tests and visual inspection of the histograms. To test a significant separation from the normality of the variables' distributions, the Lilliefors tests were applied. The Log-transformation successfully rendered the data normal in some variables. For other variables that remain not normal, a rank transformation was applied instead to conform to the use of parametric statistical models (Conover and Iman, 1981). Comparisons of clinical and demographic variables between groups were performed with Analysis of Variance (ANOVA), Kruskal-Wallis, or Chi-square tests where appropriate.

Baseline network topological measures were compared between groups with Analysis of Covariance statistical models (ANCOVA) to estimate the two main effects ApoE4 status (Carriers vs. non-Carriers) and disease progression (Converters vs. non-Converters) and the interaction term ApoE4*progression. The additional covariates were age, gender, educational level, MRI magnetic field strength, handedness, and gray matter volume. We checked whether the dependent variable's variance is equal between the groups by performing Levene's test of equal variances. If significant differences were found (all p > 0.05), either a 'rank' or a 'log10′ transformation was applied to the dependent variable. Additionally, we used a Games-Howell method when equal group/level variances are not assumed and posthoc comparisons with Tukey's or Bonferroni corrections to adjust for multiple comparisons. These statistical verifications are essential to ensure the dependent variable transformations' reliability and results’ validity using parametric models.

As reported previously, the size, degree, and connectivity density might influence other network properties (van Wijk et al., 2010, Zalesky et al., 2010). Therefore, we first tested group effects for these network properties defining size, connectivity density, and average degree. For those significant, these properties were added as additional covariates (Dicks et al., 2018, Tijms et al., 2013a)

ANCOVA analysis was performed for the normalized clustering index at each brain region defined in the Neuromorphometric atlas. We also included the regional gray matter volume and nodal degree as additional covariates. False discovery rate (FDR) correction was used to adjust for multiple comparisons by the number of structures.

The association of network properties with psychological (ADNI-MEM, MMSE) and CSF variables (Aβ42, T-tau, and P-tau) were assessed using a partial correlation model. The method allows calculating the linear partial correlation between our variables of interest adjusting for different covariates. Our covariates were: age, gender, educational level, and TIV. Where appropriate, we adjust for multiple comparisons using FDR correction.

To find statistical differences between partial correlation coefficients, we first applied the Fisher's Z transform z = ln((1 + r)/(1-r))/2 for each correlation coefficient. After, we used the Z-test correcting for degrees of freedom as follows: Z = (z1-z2)/sqrt (1/(n1-q-3) + 1/(n2-q-3)) (Afifi et al., 2003) (assuming standard normal distribution under the null hypothesis of no difference in 'mean' partial correlations); where 'z1′ and 'z2′ are the two transformed partial correlations, 'n1′ and 'n2′ are sample sizes, and 'q' is the number of covariates involved in the partial correlation computation.

The statistical analysis was performed using the JASP software (https://jasp-stats.org/). For the partial correlation analysis, we used MATLAB software ('*partialcorri.m*' function) (https://www.mathworks.com/).

### 2.8 Study of the SSGMNets reliability

To evaluate the reliability of the *SSGMNets* topological properties, we followed the methodology used in Pizzagalli et al. 2020 (Pizzagalli et al., 2020). Specifically, we selected the Kennedy Krieger Institute (KKI) (Multi-Modal MRI Reproducibility Resource) (Landman et al., 2011) data and the Intraclass correlation measure (ICC) (Landis and Koch, 1977) for the reliability evaluation. The KKI (Kennedy Krieger Institute—Multi-Modal MRI Reproducibility Resource) data is available at https://www.nitrc.org/projects/multimodal/. More details related to MRI data acquisition and processing can be found in Supplementary Materials.

## 3 Results

### 3.1 Sample description statistics and SSGMNets reliability

The groups did not significantly differ in age, gender, education, or network size (number of nodes) at time points 1 and 2 (see Table 1). All *SSGMNets* followed a small-world topology and did not exhibit disconnected nodes. We found a good/excellent reproducibility for all *SSGMNets* global network attributes with ICC > 0.8. Tables with all ICC values and the statistical significance, including the nodal normalized clustering index, can be found in Supplementary Material, Tables S26, and S27.

### 3.2 Baseline effects of ApoE4 and disease progression on the network properties

All groups showed a small-world architecture (i.e., σ >1). We found significant group differences in disease progression and ApoE4 factors in the ANCOVA analysis after correcting for multiple hypotheses. The independent effect of the ApoE4 (Carriers vs. non-Carriers) was significant in Clux-Normalized *(F(1,190) = 4.372, p = 0.038, effect-size ω^2^ = 0.014)* and σ *(F(1,190) = 4.743, p = 0.031, effect-size ω^2^ = 0.012)*.

**Fig. 1:**
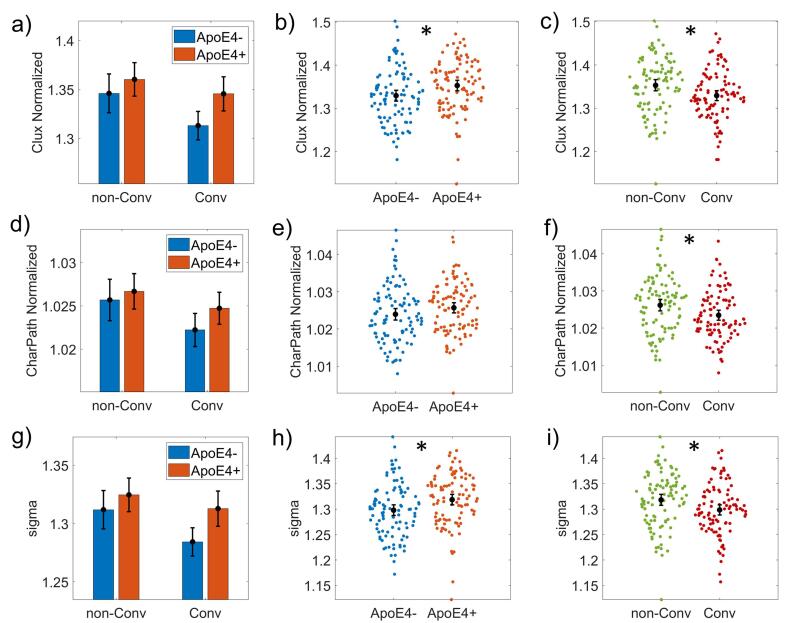
ANCOVA results for Network topological attributes. It is represented the plots of Clustering index normalized (Clux-Normalized), Characteristic path length normalized (CharPath-Normalized), and sigma. The first column of graphs (panels a), d) and g)) shows the groups subdivided into non-Converters (non-Conv), Converters (Conv), and Carriers (ApoE4 + ), non-Carriers (ApoE4-). The second column (panels b), e) and h)) shows the results of the ApoE4 main effect comparing ApoE4 + versus ApoE4-. The third column (panels c), f) and i)) shows the disease progression's results as comparing non-Converters (non-Conv) versus Converters (Conv). Asterisks indicate statistically significant differences. The bars' height represents the mean, and the error bars the 95% confidence interval (CI). Dots represent subject measures.

Fig. 1 (panels b) and h)) shows higher Clux-Normalized and σ values for Carriers than non-Carriers. The disease progression effect (Fig. 1, panels c), f), and i)) was found on Clux-Normalized *(F(1,190) = 5.053, p = 0.026, ω^2^ = 0.014),* CharPath-Normalized *(F(1,190) = 5.430, p = 0.021, ω^2^ = 0.017)* and σ *(F(1,190) = 4.618, p = 0.033, ω^2^ = 0.012)*.

MCI Converters showed lower values in all network properties than non-Converters (Fig. 1, panels c), f), i)). We did not find ApoE4*disease progression interaction effects. More details about these results can be found in Table S4, Supplementary Material.

### 3.3 ApoE4 and disease progression effects on the network properties rate of change

**Fig. 2:**
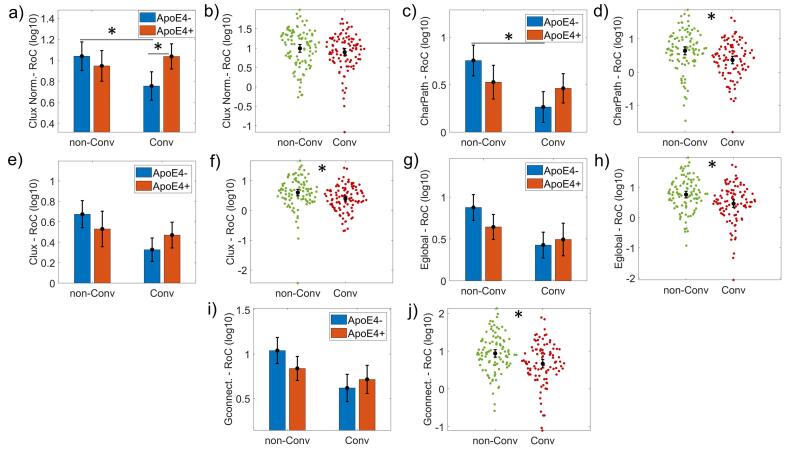
ANCOVA results for the Rate of Change (RoC) of the Network topological attributes. It is represented the bar plots of Clustering index normalized (Clux Normalized), Characteristic path length (CharPath), Clustering index (Clux), global efficiency (Eglobal), and Global connectivity (Gconnect). Panels a), c), e), g) and i) show the groups subdivided into non-Converters (non-Conv), Converters (Conv), and Carriers (ApoE4 + ), non-Carriers (ApoE4-). Panels b), d), f), h) and j) represent the disease progression's main effect results as comparing non-Converters (non-Conv) versus Converters (Conv). 'Clux Normalized' and 'CharPath' showed an interaction effect ApoE4*disease progression. All topological network attributes depicted a significant disease progression effect. A log10 transformation was applied to all variables to meet the equal group variances condition in the parametric ANCOVA statistical design. Asterisks indicate statistically significant differences (p-corrected < 0.05). The bars' height represents the mean, and the error bars the 95% confidence interval (CI). Dots represent subject measures.

We found differences (Fig. 2) in the rate of change (RoC) related to progression factor in Clux *(F(1,190) = 5.273, p = 0.023, ω^2^ = 0.021)*, CharPath *(F(1,190) = 10.378, p = 0.002, ω^2^ = 0.044),* GConnect *(F(1,190) = 10.153, p = 0.002, ω^2^ = 0.043)* and Eglobal *(F(1,190) = 10.712, p = 0.001, ω^2^ = 0.046).* The ApoE4*disease progression interaction effect was found in Clux-Normalized *(F(1,190) = 7.414, p = 0.007, ω^2^ = 0.031)* and CharPath *(F(1,190) = 5.566, p = 0.019, ω^2^ = 0.222)* Fig. 2, panels a) and c)*.* The highest interaction size effect was found in the CharPath (*size-effect ω^2^ = 0.222)*.

Interestingly, the post hoc analysis shows that only in the non-Carriers group, non-Converters showed higher Clux-Normalized and CharPath RoC than Converters. On the other hand, in the Converters group, the Carriers depicted significantly more Clux-Normalized as compared with non-Carriers. The ApoE4 effects, as a global factor, were not present in this analysis. Details about this analysis can be found in Supplementary Material Table S6.

### 3.4 Effects of ApoE4 and disease progression on cognitive, morphometric, and CSF-derived measures in baseline

**Fig. 3:**
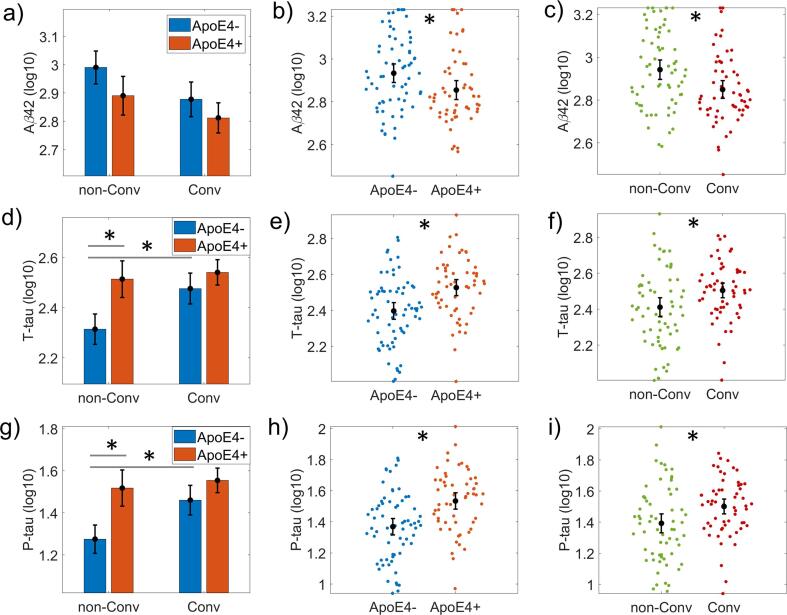
ANCOVA results for Cerebrospinal Fluid (CSF) variables. Bar plots for Aβ42, T-tau, and P-tau variables. The first column of graphs (panels a), d) and g)) shows the groups subdivided into non-Converters (non-Conv), Converters (Conv), and ApoE4+, ApoE4-. The second column (panels b), e) and h)) represents the ApoE4 main effect results comparing ApoE4 + versus ApoE4-. The third column (panels c), f) and i)) shows the disease progression's results as comparing non-Converters (non-Conv) versus Converters (Conv). The CSF measures were log10 transformed to meet the equal group variances condition in the parametric ANCOVA statistical design. Asterisks indicate statistically significant differences (p-corrected < 0.05). The bars' height represents the mean, and the error bars the 95% confidence interval (CI). Dots represent subject measures.

**Fig. 4:**
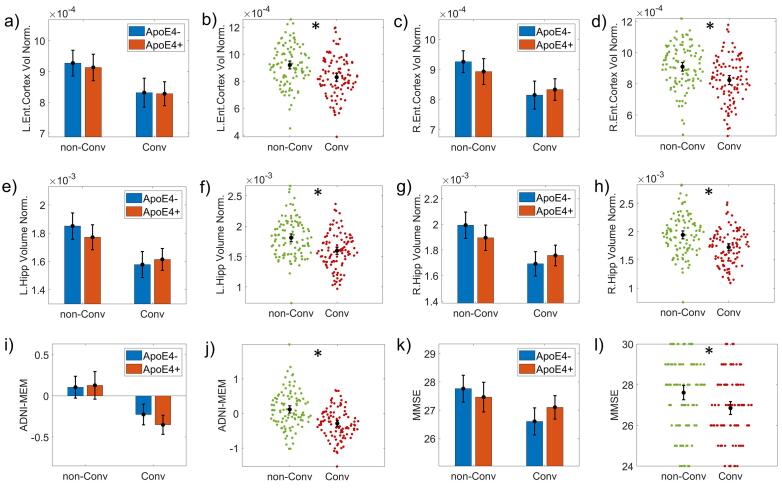
ANCOVA results for the volumetric and cognitive variables. The volumetric variables were: normalized volume of the left Entorhinal cortex (L.Ent.Cortex Vol Norm.) (panels a) and b)), and right Entorhinal cortex (R.Ent.Cortex Vol Norm.) (panels c) and d)); the normalized volume of the left Hippocampus (L.Hipp Volume Norm.) (panels e) and f)), and right Hippocampus (R.Hipp Volume Norm.) (panels g) and h)). The cognitive variables are ADNI-MEM (panels i) and j)) and Mini-Mental score (MMSE) (panels k) and l). For all variables, a bar plot with the groups subdivided in non-Converters (non-Conv), Converters (Conv), and ApoE4+, ApoE4- are shown. All variables showed a significant disease progression main effect as comparing non-Converters (non-Conv) versus Converters (Conv) (see Materials and Methods section). Asterisks indicate statistically significant differences (p-corrected < 0.05). The bars' height represents the mean, and the error bars the 95% confidence interval (CI). Dots represent subject measures.

Fig. 3, Fig. 4 show the ANCOVA results for the baseline groups' differences in cognitive, regional gray matter volume, and CSF biomarkers.

The CSF-derived measures (Aβ42, T-tau and P-tau) showed significant group differences in the ANCOVA analysis for ApoE4, disease progression and the ApoE4*disease progression interaction.

The results revealed an ApoE4 effect on Aβ42 *(F(1,118) = 7.365, p = 0.008; ω^2^ = 0.047)*; T-tau *(F(1,118) = 17.734, p = 0.001, ω^2^ = 0.110)* and P-tau *(F(1,118) = 21.739, p = 0.001, ω^2^ = 0.133)*. The MCI Carriers’ group exhibited lower CSF Aβ42 levels and higher T-tau and P-tau compared to non-Carriers at baseline.

For the disease progression factor all measures were significantly different between Converters and non-Converters (Aβ42: *F(1,118) = 9.905, p = 0.002, ω^2^ = 0.065; T*-tau: *F(1,118) = 9.473, p = 0.003, ω^2^ = 0.056); P*-tau: *(F(1,118) = 9.924, p = 0.002, ω^2^ = 0.057).*

The ApoE4*disease progression interaction was found only in Tau measures (T-tau: *F(1,118) = 4.899, p = 0.029, ω^2^ = 0.026;* P-tau: *F(1,118) = 4.522, p = 0.036, ω^2^ = 0.023).* The post hoc analysis showed significant differences between Converters and non-Converters in the non-Carriers group and between Carriers and non-Carriers in the non-Converters group. Interestingly we did not find significant differences for MCI Carriers associated with disease progression (see Supplementary Material Table S4).

We found significant effects associated with disease progression in MMSE *(F(1,194) = 14.133, p = 0.001, ω^2^ = 0.058)* and ADNI-MEM *(F(1,194) = 43.209, p = 0.001, ω^2^ = 0.166)*. The test scores showed significant differences between Converters and non-Converters in the non-Carriers group (see Supplementary Material Table S4). ADNI-MEM also captured this difference for the Carrier’s group. Finally, the left and right hippocampus and entorhinal gray matter volume normalized revealed differences between non-Converters and Converters (*R.Hipp*: *(F(1,191) = 25.482, p = 0.001, ω^2^ = 0.091; L.Hipp: (F(1,191) = 28.141, p = 0.001, ω^2^ = 0.099; R.EC: (F(1,191) = 17.803, p = 0.001, ω^2^ = 0.069; L.EC: F(1,191) = 17.537, p = 0.001, ω^2^ = 0.070)* (Supplementary Material Table S4). However, both right hemisphere regions, showed differences associated with disease progression for non-Carrier’s group. In the left hemisphere significant differences were also found for Carriers.

The highest size effects associated with ApoE4 was found for P-tau (*ω^2^ =* 0.13) and T-tau (*ω^2^ =* 0.11). The ADNI-MEM showed the maximum size effect associated with the disease progression (*ω^2^ = 0.166)*.

The post hoc testing results (p-value corrected by Bonferroni) for all variables with statistically significant differences can be found in Supplementary Material Table S4.

### 3.5 ApoE4 and disease progression effects on the rate of change of cognitive and morphometric variables

**Fig. 5:**
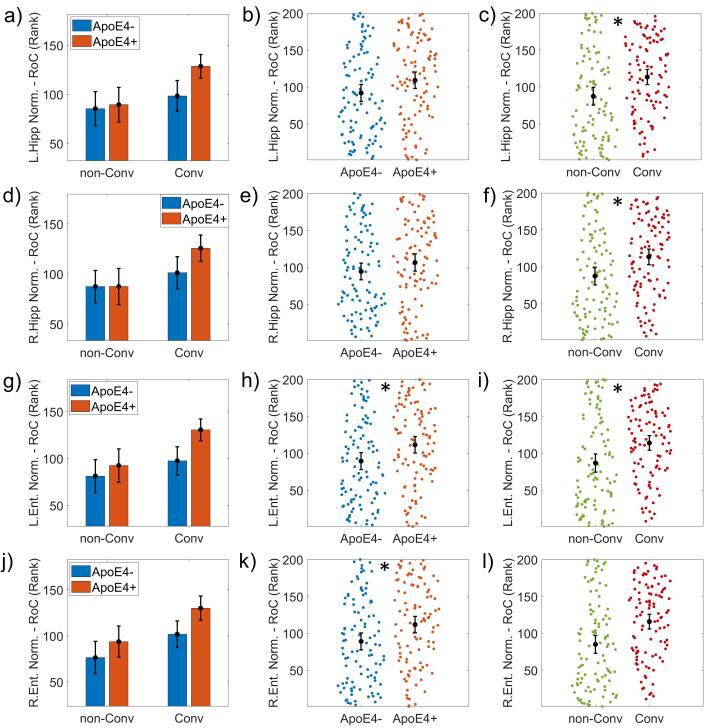
ANCOVA results for the Rate of Change (RoC) of the volumetric measures. It is represented the bar plots of the normalized volume of the left Hippocampus (L.Hipp Volume Norm.) (panels a), b) and c)), right Hippocampus (R.Hipp Volume Norm.) (panels d), e) and f)) and the normalized volume of the left Entorhinal cortex (L.Ent.Cortex Vol Norm.) (panels g), h) and i)), and right Entorhinal cortex (R.Ent.Cortex Vol Norm.) (panels j), k) and l)). The first column of graphs shows the groups subdivided into non-Converters (non-Conv), Converters (Conv), and APOE4+, APOE4-. The second column shows the results of the APOE4 main effect comparing APOE4 + versus APOE4-. The third column shows the disease progression's main effect as comparing non-Converters (non-Conv) versus Converters (Conv). 'L.Ent.Cortex Vol Norm.' and 'R.Ent.Cortex Vol Norm.' showed a significant APOE4 effect. A 'rank' transformation was applied to meet the equal group variances condition in the parametric ANCOVA statistical design. Asterisks indicate statistically significant differences (p-corrected < 0.05). The bars' height represents the mean, and the error bars the 95% confidence interval (CI). Dots represent subject measures.

The normalized volume in left and right entorhinal cortex showed groups significant differences associated with the disease progression (*F(1,191) = 10.869, p < 0.00, ω^2^ = 0.046; F(1,191) = 14.823, p < 0.001, ω^2^ = 0.064)* as well as in the right and left hippocampus (*F(1,191) = 9.573, p = 0.002, ω^2^ = 0.041; F(1,191) = 9.271, p = 0.003, ω^2^ = 0.039)* (see Fig. 5)*.*

**Fig. 6:**
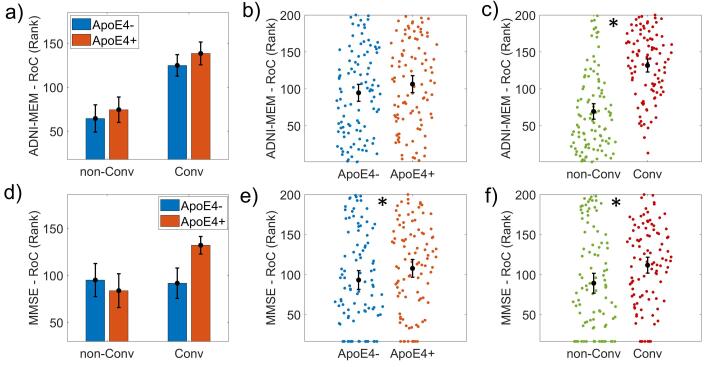
ANCOVA results for the Rate of Change (RoC) of cognitive variables. It is represented the bar plots of the cognitive variables are ADNI-MEM (panels a), b) and c)) and Mini-Mental score (MMSE) (panels c), d) and e)). The first column of graphs shows the groups subdivided into non-Converters (non-Conv), Converters (Conv), and APOE4+, APOE4-. The second column shows the results of the APOE4 main effect comparing APOE4 + versus APOE4-. The third column represents the disease progression's main effect, comparing non-Converters (non-Conv) versus Converters (Conv). Both variables showed a significant disease progression effect. MMSE also showed a significant APOE4 effect (panel e)). A 'rank' transformation was applied to meet the equal group variances condition in the parametric ANCOVA statistical design. Asterisks indicate statistically significant differences (p-corrected < 0.05). The bars' height represents the mean, and the error bars the 95% confidence interval (CI). Dots represent subject measures.

We found an ApoE4-related effect on the rate of change of MMSE *(F(1,194) = 62.744, p < 0.001, ω^2^ = 0.015)*, the left and right entorhinal cortex normalized volume *(F(1,191) = 8.236, p = 0.007, ω^2^ = 0.034; F(1,191) = 8.616, p = 0.004, ω^2^ = 0.064)*. The MMSE and ADNI-MEM rate of change were also affected by the disease progression *(F(1,194) = 62.744, p < 0.001, ω^2^ = 0.231; F(1,194) = 77.778, p < 0.001, ω^2^ = 0.278)* (see Fig. 6)*.*

The ApoE4 highest size effect was found for the right entorhinal cortex (*ω^2^ =* 0.06). The MMSE (*ω^2^ =* 0.231), ADNI-MEM (*ω^2^ = 0.278),* and right entorhinal cortex *(ω^2^ = 0.06)* showed the maximum size effects associated with the disease progression. The results of the posthoc comparison can be found in Supplementary Material Table S6.

### 3.6 ApoE4 and disease progression-related changes on regional normalized clustering index

As the network Clux-Normalized showed significant group differences associated with the ApoE4 and disease progression factors and its RoC, we studied the origin of these differences at the regional level.

The normalized clustering index is commonly used as an indicator of functional segregation and, as such, suggests the role of a particular region on specialized processing that occurs within densely interconnected groups of brain regions.

We found 13 regions with differences between Carriers and non-Carriers (p-uncorrected < 0.01) (Supplementary Material Table S8). However, only the right supramarginal gyrus – PCgG.R – (p_FDR_ = 0.043) and the left anterior cingulate gyrus – ACgG.L – (p_FDR_ = 0.015) survived multiple comparison correction. Both showed higher Clux-Normalized values for Carriers compared to non-Carriers.

**Fig. 7:**
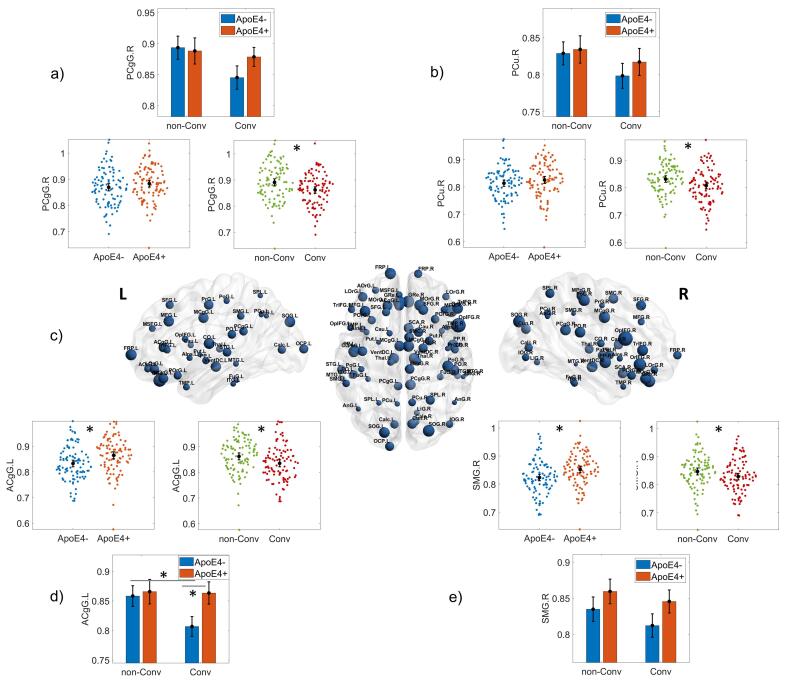
ANCOVA results for the nodal normalized clustering index (nodal Clux Normalized) topological measure. All structures represented as spheres in panel c) were those with significant p-values (FDR corrected multiple comparisons) for the disease progression main effect (Converters versus non-Converters). The larger the sphere diameter, the larger the difference between groups. The panels a) and b) represent the results for the right Posterior Cingulate gyrus (PCgG.R) and the right Precuneus (PCu.R), respectively. Panel d) and e) show the left Anterior Cingulate gyrus (ACgG.L) and right Supramarginal gyrus (SMG.R), respectively. These structures showed differences in ApoE4 main effect (p uncorrected). The ACgG.L also shows an interaction effect of APOE-disease progression. Asterisks indicate statistically significant differences for p-corrected < 0.05 (FDR) except for panel d) and e) p-uncorrected < 0.01. The bars' height represents the mean, and the error bars the 95% confidence interval (CI). Dots represent subject measures.

**Fig. 8:**
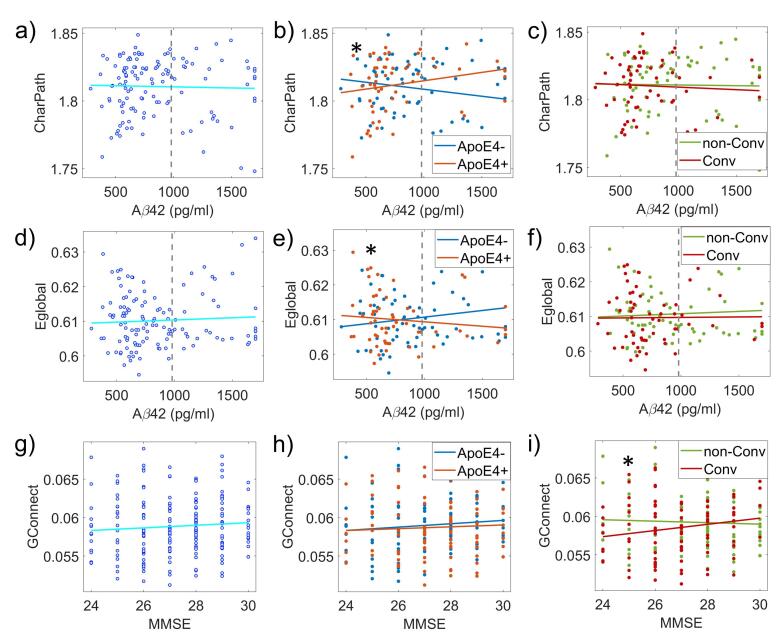
Differences in the correlation between Network topological attributes and cognitive and CSF variables: APOE4 and disease progression effects. Significant differences between APOE4 + versus APOE4- in the correlation of the variables Characteristic path length (CharPath) (panel b) and Global Efficiency (Eglobal) (panel e)) with Aβ42. Global connectivity (GConnect) correlation with MMSE is different between Converters (Conv) and non-Converters (non-Cov) (panel i)). Gray dotted lines in Aβ42 plots denote this parameter's normality limit (980 pg/ml). Asterisks indicate statistically significant differences (p < 0.05).

For the disease progression effect, 75 regional ANCOVAs were significantly different between groups, after FDR correction for multiple comparisons (Supplementary Material Table S9). Fig. 7 shows the spatial distribution of regional normalized clustering index group differences. The MCI that will progress into AD showed lower Clux-Normalized for all regions than those that will not. Some of these regions were: right fusiform gyrus (p_FDR_ = 0.006), right and left middle frontal gyrus (p_FDR_ = 0.002; p_FDR_ = 0.0001), right and left posterior cingulate cortex (p_FDR_ = 0.002; p_FDR_ = 0.005), right precuneus (p_FDR_ = 0.006), right and left superior frontal gyrus (p_FDR_ = 0.0001; p_FDR_ = 0.001) and left supramarginal gyrus (p_FDR_ = 0.005). For details of regional groups' mean and confident intervals, see Supplementary Material Table S9. (See Fig. 8)

The left anterior cingulate gyrus (ACgG.L) showed significant interaction effects ApoE4*disease progression but for p-uncorrected < 0.01.

### 3.7 ApoE4 and disease progression effects on the association between network properties and cognitive and CSF variables

We further scrutinized the relationships between network measures, memory deficit measured with ADNI-MEM, cognitive decline evaluated with MMSE (available for all subjects), as well as CSF Aβ42, T-tau, and P-tau levels.

The analysis pulling together all groups and at individual groups showed several associations between the network properties and other measures (Supplementary Material Tables S10–S14).The Clux-Normalized was related to ADNI-MEM using all subjects (r = 0.206, p = 0.003) and with Carriers (r = 0.225, p = 0.03) and non-Carriers (r = 0.234, p = 0.021) groups separated. The clustering index correlated with ADNI-MEM in general (r = 0.259, p = 0.0002, N = 200 subjects), non-Converters (r = 0.249, p = 0.014, N = 100), as well as with MMSE in the non-Carriers (r = 0.217, p = 0.033, N = 100). Also, clustering index showed a correlation with ADNI-MEM in non-Carriers group (r = 0.388, p = 8.97*10^−5^, N = 100) and not in Carriers.

We found the normalized characteristic path length (CharPath-Normalized) to correlate in general with ADNI-MEM (r = 0.246, p = 0.0005, N = 200). In Carriers, CharPath-Normalized was associated with ADNI-MEM (r = 0.297, p = 0.003) and in non-Carriers (r = 0.232, p = 0.023) as well. Finally, CharPath-Normalized show significant correlations with MMSE in non-Carriers (r = 0.148, p = 0.038) and with Aβ42 in Carriers (r = 0.283, p = 0.039).

The global connectivity showed association with ADNI-MEM in general (r = 0.162, p = 0.024), non-Converters (r = 0.24, p = 0.016) and non-Carriers (r = 0.287, p = 0.004) groups. Finally, in non-Converters, we found an association between global efficiency and ADNI-MEM (r = 0.209, p = 0.04). The CSF Tau measures did not reveal associations with the network properties.

For ApoE4, we found significant differences in the correlations between the characteristic path length and Aβ42 levels (Carriers r = 0.229, non-Carriers r = -0.171, z-stats = 2.158, p = 0.03). Also in global efficiency we obtained the same effect (Carriers r = -0.16, non-Carriers r = 0.20, z-stats = -2.00, p = 0.044).

The correlation between global connectivity and MMSE was different between Converters and non-Converters (Converters r = 0.2, non-Converters r = -0.08, z-stats = 2.01, p = 0.04). The correlation between network properties with ADNI-MEM and CSF tau measures did not reveal significant differences between Converters versus non-Converters and Carriers versus non-Carriers.

## 4 Discussion

The present study shows that *SSGMNet* is affected independently by ApoE4 and disease progression in late-MCI. The topological network alterations indicate a shift towards a random organization, more in Carriers than non-Carriers. Our findings reveal the intricate relationship between the *SSGMNet* attributes and ApoE4 genotype, suggesting modulated effects by independent processes associated with disease progression.

The main results of this research can be summarized as follows: 1) At baseline (all subjects classified as MCI) the ApoE4 and the future progression to AD status modulate topological network properties differently; 2) the Rate of Change (RoC) of characteristic path length and Clux-Normalized were affected by ApoE4 and disease progression status interaction; 3) The Clux-Normalized values were lower in MCI who will progress into AD compared to those who will not; 4) Clux-Normalized decreased in several regions belonging to the Default Mode Network (DMN) in MCI Converters respect to non-Converters; 5) ApoE4 and disease progression affect the association between specific topological network features and CSF Aβ42; and MMSE variables.

The present results are in line with the idea that disruptions in gray matter networks start years before dementia unfolds and, as such, may be sensitive to the concurrent changes in structural integrity across the brain in MCI. The baseline and RoC analysis findings underline the importance of considering cross-sectional and longitudinal approaches, as they could provide complementary information. Some of these findings deserve more attention and will be discussed in the following subsections.

### 4.1 ApoE4 and progression to AD status impairs the SSGMNet topology in MCI

Our results revealed that the ApoE4 mainly modulated two network properties, Normalized clustering index, and sigma. In both attributes, higher values in MCI Carriers were found than non-Carriers independently of the disease progression status. These increments (e.g., higher similarity between neighboring nodes) associated with the E4 allele may reflect synchronous atrophy between brain areas, whereas Carriers show a more uniform neurodegeneration pattern across the brain. This finding seems to contradict previous studies reporting lower clustering index in MCI Carriers (Li et al., 2019, Sanabria-Diaz et al., 2021). The source of this variability may be primarily related to a different network methodology, morphometric descriptors, group selection, and sample size, among other factors.

Additionally, the higher values of small-worldness in Carriers indicate a less random network organization, which has often been reported in AD and MCI (Dicks et al., 2018, Tijms et al., 2013a, Yao et al., 2010). The disease progression also modulated the sigma property, with lower values in Converters compared to non-Converters. This association has been demonstrated in previous longitudinal and cross-sectional studies in MCI (Friedman et al., 2014).

Independent of the ApoE4 factor, we found that those MCI who will progress into AD have lower normalized characteristic path length. It suggests a more random network, consequently reducing the potential for functional integration between brain regions. This effect potentially reflects the interplay between synchronous atrophy over time (Tijms et al., 2018) and regional adaptative/maladaptive mechanisms (Fornito et al., 2015).

Additionally, only the CSF measures were modified by the E4 allele at baseline. Our results in Carriers confirm previous findings where ApoE4 status was associated with brain amyloid accumulation and lower CSF Aβ42 as well as higher tau levels in MCI (Hashimoto et al., 2012; Liu et al., 2013; Risacher et al., 2013). Yet, the impact of the disease progression status at baseline was captured by the hippocampus and entorhinal volumes and ADNI-MEM composite score. The MCI Converter groups showed higher volume loss in both structures and lower scores on the memory test than the non-Converters. Both measures have been previously associated with MCI progression into AD (Crane et al., 2012, Farlow et al., 2004, Giorgio et al., 2020; Jack et al., 2010a; Jack et al., 2013, Jack et al., 2004).

Compared to our previous paper (Sanabria-Diaz et al., 2021), there are several differences. Contrary to the mentioned study, we also classified MCI patients at baseline based on the clinical progression (non-Converters versus Converter into AD). Second, the evaluation of the network measures is different between studies. We found the Clux-Normalized more sensitive to detect ApoE4 effects, a network attribute that was not explored in the previous work. Each research used a different morphometric descriptor (cortical thickness versus grey matter density). Based on a prior study from our group, it is known that morphometric descriptors capture distinct properties of the interaction between brain structures (Sanabria-Diaz et al., 2010). Importantly, we explore the modulation on the time Rate of Change (RoC) of network attributes, CSF biomarkers, and cognitive measures in the same MCI cohort. This relevant contribution is missing in our previous paper.

### 4.2 ApoE4 genotype differentially modulates the rate of change of SSGMNet properties and other AD-related biomarkers

Our study revealed that the RoC of characteristic path length and Normalized clustering index were affected by the interaction between ApoE4 and disease progression status. The effect was driven by the non-Carrier's group, were patients who will later on progress into AD showed the steepest decline compared to those that will not convert to AD. This result may help to establish which network properties changes are associated with AD progression in MCI non-Carriers. It supports the hypothesis that a higher rate of decreasing over time in both metrics in MCI is associated with an AD progression. In particular, a previous study in non-demented subjects (amyloid positive) showed an association between clinical progression over time and lowered normalized clustering index values (Tijms et al., 2016). Also, RoC results indicated that the gray matter networks seem to move towards a random network organization, which has been previously reported for AD subjects by other studies (Pereira et al., 2016, Phillips et al., 2015, Tijms et al., 2014 (Tijms et al., 2013a)).

Interestingly, none of the network measures’s RoC is affected by ApoE4 as a global ANOVA effect, although several were sensitive to the disease progression. Longitudinally, the main differences associated with the clinical progression were found to have a steeper decrease in cluster index, characteristic path length, global connectivity, and global efficiency in Converters. Altogether these findings suggest brain connectivity alterations (for a review, see (Tijms et al., 2013b)). They may reflect a reduced ability to integrate information across distributed brain regions and altered communication between neighboring areas (Dicks et al., 2018, Pereira et al., 2016).

Additionally, we found an independent effect of the E4 allele on the entorhinal cortex rate of atrophy. The Carriers showed a higher RoC in this structure volume compared to non-Carriers. Specifically, a previous study using ADNI database confirms an association between E4 allele and a more significant increase in atrophy rate in the hippocampus and entorhinal cortex in MCI Carriers' Converters (Hostage et al., 2014, Risacher et al., 2010). In our study, the steeper atrophy in these regions in MCI Converters confirms the most extensive effects described in areas previously demonstrated to display significant atrophy in AD. Nevertheless, this analysis revealed the importance of monitoring the entorhinal cortex volume in MCI since it is affected independently by ApoE4 and disease progression.

A faster cognitive decline associated with ApoE4 was also captured by MMSE. Our result suggests that this test score, used in clinical and research settings to measure cognitive impairment, is modulated by the ApoE4. Based on this finding, we considered incorporating the subject E4 allele information for MCI cognitive characterization a valuable research strategy, especially in clinical trials. On the other hand, independently of ApoE4, the RoC for ADNI-MEM total score revealed faster memory decline for those who progressed into AD. ADNI-MEM has been considered in a previous study using the ADNI database, as the most discriminative cognitive feature for classifying stable versus progressive MCI (Giorgio et al., 2020).

### 4.3 ApoE4 and disease progression modulate the regional Normalized clustering index in MCI

Our study found regional normalized clustering index differences between groups driven by the disease progression and not by ApoE4. These regional differences were widely distributed across the brain with a common denominator: lower Clux-Normalized values for those MCI that will convert to AD. It suggests that in MCI, the risk of disease progression is characterized by worse local communication between 'topological neighboring' areas (graph theory concept). The fact that all identified regions showed a lower clustering index in MCI Converters may be related to AD neuropathological processes that are already operating in this phase, which in turn affect the intracortical gray matter properties similarity.

It has been previously described that intracortical morphometric similarity is related to coordinated changes of cortical structures (for a review, see (Alexander-Bloch et al., 2013)). This hypothesis may explain why several regions with lower values in MCI Converters belong to the default-mode network (DMN), including the posterior cingulate cortex, precuneus, temporal and prefrontal areas (Greicius et al., 2004). This finding agrees with our previous study, where several regions belonging to the DMN showed nodal alteration associated with the ApoE4 (Sanabria-Diaz et al., 2021). However, many of them diverge between studies probably related to several methodological differences (i.e., parcellation schemes and sample size).

Our findings support the idea that a continuous DMN activity increases the metabolism-dependent cascade conducive to AD (Buckner et al., 2009, Buckner et al., 2005, Raichle, 2006). In this regard, as part of the DMN, memory systems may be preferentially affected because it plays a central role in the resting activity (Buckner et al., 2005).

In addition to this hyper functional-activation hypothesis, the regional clustering alterations may arise due to axonal connectivity. For example, we found lower values in the posterior cingulate cortex and the precuneus for those MCI who will progress into AD. Both structures have been reported in previous network studies in MCI and AD at a group and individual-based level (Dicks et al., 2018, He et al., 2008, Pereira et al., 2016, Tijms et al., 2014, Tijms et al., 2013a, Yao et al., 2010). Moreover, the posterior cingulate cortex constitutes a central node in the DMN. This structure has reciprocal connections to the medial temporal lobe structures (i.e., entorhinal cortex, parahippocampal gyrus, precuneus, orbitofrontal cortex) affected during AD progression. Along with the precuneus, the posterior cingulate cortex has a role in episodic memory retrieval (Maddock et al., 2001, Nielsen et al., 2005), working memory (Kozlovskiy et al., 2012), and has been implicated in several intrinsic control networks (Leech et al., 2012).

These results support the hypothesis that in MCI, regional network alterations are associated with network degeneration. There are several mechanisms suggested by which it occurs: 1) Selective neuronal vulnerability that may affect functional circuits (Hyman et al., 1984), inducing compensatory strategies at the network level (Palop et al., 2007, Palop et al., 2006); 2) Retrograde axonal transport deficits that result in axonal degeneration (Salehi et al., 2006); 3) Prion disease mechanism where misfolded disease proteins may propagate throughout brain circuits (Scott et al., 2013).

### 4.4 ApoE4 and disease progression effects on the association between network properties, cognitive and CSF-derived measures

Additionally, the present study investigated the associations between the network topological properties, memory, general cognition, and CSF-derived measures. We studied the general correlation between these variables and, separately, the influence of ApoE4 and disease progression factors. We found that in MCI, several network properties showed a positive correlation with memory functions evaluated using ADNI-MEM. Our findings agree with a recent MCI study in which the gray matter network properties showed the strongest associations with a decline in global cognition and memory (Dicks et al., 2018). The correlation with ADNI-MEM is consistent with the memory domain being among the first cognitive functions affected in the amnesic MCI subtype (Jack et al., 2013). A practical implication of this finding is the possible use of the network measures to monitor new therapies' efficiency in clinical trials in MCI patients.

Interestingly, the CSF Aβ42 level was positively associated with the characteristic path length in the Carriers group, while we did not find correlations with P-tau and T-tau. This result agrees with previous studies showing that the E4 allele modulates the brain Aβ aggregation and deposition (for review, see (Liu et al., 2013)). Moreover, the positive association between these two measures was confirmed only for ApoE4 and not for the disease progression factor. Our finding suggests that the characteristic path length informs the detrimental E4 allele effects on the amyloid-related pathways in network terms. We hypothesized that synaptic dysfunction due to increased AD-related brain pathology (amyloid aggregation, tau) renders gray matter morphology more dissimilar at a regional level, resulting in differences between Carriers and non-Carriers.

Finally, the CSF P-tau and T-tau levels were not associated with the network topology either for ApoE4 or disease progression factors. This finding may suggest that the network attributes are not sensitive to Tau levels changes and/or may reflect a different neurodegenerative mechanism. Consistent with this explanation, a previous study found gray matter network measures contained predictive information in addition to total CSF Tau levels (Tijms et al., 2018).

### 4.5 Limitations and Future considerations

There are several potential limitations to this study that should be considered. First, two-time points MRI scans were analyzed in the current study. Future investigation on the ApoE4-related effects on the network properties, longitudinal cognitive decline, and brain atrophy is necessary. Second, a small sample of subjects had CSF measures, which might have affected the accuracy of the association analysis. Third, this study was specifically limited to those subjects who were already clinically diagnosed with amnesic MCI. Thus, our study may not be generalizable to other clinical studies or populations. Further studies are needed to support the present findings with larger sample sizes.

A gene-dose analysis as well as susceptibility and protective loci associated with late-onset AD need to be considered in conjunction to ApoE4 for studying possible interaction effects. Another potential limitation is that our study included an average period of 3 years between the two visits (time points). Hence, some patients in the non-Converter MCI group could progress to dementia later on. Yet, we demonstrated the impact of the E4 allele on the RoC of structural gray matter networks alongside the cross-sectional results. To the best of our knowledge, this has not been studied before and warrants further investigation of how gray matter network integrity changes over time in MCI.

Future research is needed to examine whether altered graph properties are related to a particular cognitive domain and are more sensitive to predict cognitive decline. The graphs' diagnostic potential should be further investigated using classification algorithms and state of the art machine learning algorithms.

It remains an open question how the brain structural co-variance connectivity is related to anatomical and functional connectivity and how this relationship changes during AD progression. Future multimodal neuroimaging studies are required to answer this question. A strength of the current approach is that we illustrated the network properties affected by ApoE4 and disease progression and their relation to inter-individual differences in other biomarkers in MCI, suggesting that they encode additional relevant information.

## 5 Conclusions

This paper demonstrated the role of ApoE4 in disrupting specific parameters of the gray matter network topology. ApoE4 simultaneously affects morphometric, cognitive variables, as well as CSF variables. Significantly, the time RoC of these variables is also affected by ApoE4. In particular, in Carriers, there are an increased CSF Aβ42 and entorhinal cortex atrophy and decreased T-tau and P-tau levels. We also discovered specific disruption in topological network properties, morphometric, cognitive, and CSF-derived markers in those MCI patients that will progress to AD. Disease progression conducts more pervasive brain alterations than ApoE4. The clustering index at the regional level showed widespread changes across the brain cortex, driven mostly by the disease progression, overlapping with the critical nodes of the DMN related to AD pathology.

Based on these findings, we considered the *SSGMNets* as a valid approach to sheds light on the cognition-gene-structural co-variance interaction. This is a potentially significant development because it could find use in MCI biomarkers research and may even offer clinical value. The study further provides information to advance the current understanding of how ApoE4 -which is far the most important genetic factor known in late-onset AD- influences brain network topology in MCI subjects. Examining ApoE4 with factors such as the risk of AD progression, as we have demonstrated, may be crucial in building classification models in an attempt to measure subtle network changes in MCI.

## CRediT authorship contribution statement

**Gretel Sanabria-Diaz:** Conceptualization, Data curation, Methodology, Visualization, Writing - original draft. **Jean-Francois Demonet:** Supervision, Writing - original draft. **Borja Rodriguez-Herreros:** Formal analysis, Writing - original draft. **Bogdan Draganski:** Supervision, Writing - original draft. **Ferath Kherif:** Methodology, Supervision, Writing - original draft. **Lester Melie-Garcia:** Conceptualization, Formal analysis, Methodology, Software, Supervision, Visualization, Writing - original draft.

## Declaration of Competing Interest

The authors declare that they have no known competing financial interests or personal relationships that could have appeared to influence the work reported in this paper.
