# Supplementary material for: Apolipoprotein E allele 4 effects on Single-Subject Gray Matter Networks in Mild Cognitive Impairment
Source: Neuroimage Clin. 2021 Aug 24;32:102799. doi: 10.1016/j.nicl.2021.102799 (PMC8405842; doi:10.1016/j.nicl.2021.102799)
Supplement: Supplementary data 1 [file mmc1.docx]

**Apolipoprotein E allele 4 effects on Single-Subject Gray Matter Networks in Mild Cognitive Impairment**

Gretel Sanabria-Diaz, Jean-Francois Demonet, Borja Rodriguez-Herreros, Bogdan Draganski Ferath Kherif, Lester Melie-Garcia, for the Alzheimer's Disease Neuroimaging Initiative

**Supplementary Material**

**Table S1**. List of gray matter structures defined in the Neuromorphometrics atlas.

| Structure Name | Abbreviated Name Left | Abbreviated Name Right |
| --- | --- | --- |
| Accumbens Area | Accum.R | Accum.L |
| Amygdala | Amyg.R | Amyg.L |
| Caudate | Cau.R | Cau.L |
| Hippocampus | Hip.R | Hip.L.L |
| Pallidum | Pal.R | Pal.L |
| Putamen | Put.R | Put.L |
| Thalamus Proper | Thal.R | Thal.L |
| Ventral DC | VentDC.R | VentDC.L |
| Anterior cingulate gyrus | ACgG.R | ACgG.L |
| Anterior insula | AIns.R | AIns.L |
| Anterior orbital gyrus | AOrG.R | AOrG.L |
| Angular gyrus | AnG.R | AnG.L |
| Calcarine cortex | Calc.R | Calc.L |
| Central operculum | CO.R | CO.L |
| Cuneus | Cun.R | Cun.L |
| Entorhinal area | Ent.R | Ent.L |
| Frontal operculum | FO.R | FO.L |
| Frontal pole | FRP.R | FRP.L |
| Fusiform gyrus | FuG.R | FuG.L |
| Gyrus rectus | GRe.R | GRe.L |
| Inferior occipital gyrus | IOG.R | IOG.L |
| Inferior temporal gyrus | ITG.R | ITG.L |
| Lingual gyrus | LiG.R | LiG.L |
| Lateral orbital gyrus | LOrG.R | LOrG.L |
| Middle cingulate gyrus | MCgG.R | MCgG.L |
| Medial frontal cortex | MFC.R | MFC.L |
| Middle frontal gyrus | MFG.R | MFG.L |
| Middle occipital gyrus | MOG.R | MOG.L |
| Medial orbital gyrus | MOrG.R | MOrG.L |
| Postcentral gyrus medial segment | MPoG.R | MPoG.L |
| Precentral gyrus medial segment | MPrG.R | MPrG.L |
| Superior frontal gyrus medial segment | MSFG.R | MSFG.L |
| Middle temporal gyrus | MTG.R | MTG.L |
| Occipital pole | OCP.R | OCP.L |
| Occipital fusiform gyrus | OFuG.R | OFuG.L |
| Opercular part of the inferior frontal gyrus | OpIFG.R | OpIFG.L |
| Orbital part of the inferior frontal gyrus | OrIFG.R | OrIFG.L |
| Posterior cingulate gyrus | PCgG.R | PCgG.L |
| Precuneus | PCu.R | PCu.L |
| Parahippocampal gyrus | PHG.R | PHG.L |
| Posterior insula | PIns.R | PIns.L |
| Parietal operculum | PO.R | PO.L |
| Postcentral gyrus | PoG.R | PoG.L |
| Posterior orbital gyrus | POrG.R | POrG.L |
| Planum polare | PP.R | PP.L |
| Precentral gyrus | PrG.R | PrG.L |
| Planum temporale | PT.R | PT.L |
| Subcallosal area | SCA.R | SCA.L |
| Superior frontal gyrus | SFG.R | SFG.L |
| Supplementary motor cortex | SMC.R | SMC.L |
| Supramarginal gyrus | SMG.R | SMG.L |
| Superior occipital gyrus | SOG.R | SOG.L |
| Superior parietal lobule | SPL.R | SPL.L |
| Superior temporal gyrus | STG.R | STG.L |
| Temporal pole | TMP.R | TMP.L |
| Triangular part of the inferior frontal gyrus | TrIFG.R | TrIFG.L |
| Transverse temporal gyrus | TTG.R | TTG.L |

**Table S2.** Cognitive, CSF, and morphometric measures characteristics of the MCI groups.

|  | **MCI-Non-Converters** | | | | **MCI Converters** | | | |  |  |
| --- | --- | --- | --- | --- | --- | --- | --- | --- | --- | --- |
|  | Diagnosis Time 1  (MCI) | | Diagnosis Time 2  (stable MCI) | | Diagnosis Time 1  (MCI) | | Diagnosis Time 2  (AD) | | Post Hoc Test | |
|  | carriers | non-carriers | carriers | non-carriers | carriers | non-carriers | carriers | non-carriers | Time 1 | Time 2 |
| # of Participant | 50 | 50 | 50 | 50 | 50 | 50 | 50 | 50 | na | na |
| ADNI-MEM | 0.128  (0.590) | 0.105  (0.467) | 0.090  (0.611) | 0.205  (0.653) | -0.351  (0.409) | -0.226  (0.444) | -1.189  (0.698) | -0.809  (0.568) | 2,3^***^  4,5^**^ | 1^*^  2,3,4,5  *** |
| R. Hipp volume, mm^3^ | 2.890  (0.508) | 3.006  (0.512) | 2.678  (0.597) | 2.864  (0.638) | 2.669  (0.462) | 2.558  (0.497) | 2.257  (0.533) | 2.249  (0.597) | 3,4^**^  5^***^ |  |
| L. Hipp volume, mm^3^ | 2.701  (0.453) | 2.791  (0.465) | 2.488  (0.497) | 2.655  (0.586) | 2.448  (0.438) | 2.385  (0.493) | 2.027  (0.458) | 2.084  (0.573) | 2^*^  3,4^**^  5^***^ |  |
| R. E.C volume, mm^3^ | 1.363  (1.367) | 1.398  (0.197) | 1.268  (0.258) | 1.357  (0.245) | 1.268  (0.219) | 1.233  (0.248) | 1.080  (0.256) | 1.099  (0.297) | 3,4^**^  5^***^ |  |
| L.E.C volume, mm^3^ | 1.395  (0.231) | 1.400  (0.219) | 1.293  (0.238) | 1.354  (0.255) | 1.258  (0.231) | 1.257  (0.255) | 1.065  (0.242) | 1.125  (0.293) | 2^*^  3,4^**^  5^***^ |  |
| CSF test participant (BM+: Aβ_42_, P-tau, T-tau) | 31  (22,23,21) | 33  (17,10,7) | na | na | 26  (25,23,20) | 34  (25,23,22) | na | na | Time 1 | Time 2 |
| CSF Aβ_42,_ pg/ml | 853.387  (396.721) | 1045.530  (377.282) | na | na | 681.200  (248.598) | 817.279  (342.995) | na | na | 3^***^ | na |
| CSF P-tau, pg/ml | 37.707  (20.390) | 20.744  (9.983) |  |  | 37.716  (12.372) | 31.820  (13.991) |  |  | 3,6^***^  5^**^ |  |
| CSF T-tau, pg/ml | 360.155  (161.852) | 222.791  (94.803) | na | na | 361.515  (104.888) | 323.006  (125.550) | na | na | 3,6^***^  5^*^ | na |

Legend: Aβ42: Amyloid-beta 42, P-tau: phosphorylated tau, T-tau: total tau, Hipp: hippocampus, E.C: entorhinal cortex, L: left, R: right, mm3: millimeter, pg/ml : picogram/milliliter, CSF : cefaloraquidic liquid, BM+ : biomarker positive.

Note. For ANOVA, p-value and confidence intervals adjusted using the Tukey method. For Kruskal-Wallis test, we applied the Games-Howell method, where equal group/level variances are not assumed. The p-values are corrected with the Tukey method. *p < .05; ** p < .01, ***p<0.001. Post hoc contrasts : ^1^Converter carriers - Converter non-carriers ; ^2^Converter carriers - non-Converter carriers ; ^3^Converter carriers - non-Converter non-carriers; ^4^Converter non-carriers - non-Converter carriers; ^5^Converter non-carriers - non-Converter non-carriers ; ^6^Non-converter carriers - non-Converter non-carriers

**Table S3.** MCI classification based on the ATN system.

We assigned “A+” to those individuals that had a CSF Aβ1-42< 980 pg/ml, “T+” to those individuals with P-tau > 24 pg/ml and “N+” to those individuals with T-tau > 266 pg/ml. We clustered the biomarker profiles into three categories. The A–T–N– profile was named the “normal AD biomarker” category (green shadow). We clustered the remaining A– (A–T–N+, A–T+N–, and A–T+N+) as “non-AD related pathology” (grey shadow), and all A+ (A+T–N–, A+T–N+, A+T+N–, A+T+N+) as “Alzheimer Disease-related pathology” (orange shadow).

|  | **Groups** | | | | |
| --- | --- | --- | --- | --- | --- |
| **ATN classification** | Converters  non-Carriers | Converters  Carriers | Non-converters  Carriers | Non-converters  non-Carriers | Total |
| A-T-N- | 3 | 1 | 4 | 13 | 21 |
| A-T-N+ | 1 | 0 | 0 | 1 | 2 |
| A-T+N- | 1 | 0 | 0 | 0 | 1 |
| A-T+N+ | 4 | 0 | 5 | 3 | 12 |
| A+T-N- | 7 | 3 | 4 | 11 | 25 |
| A+T-N+ | 1 | 2 | 2 | 2 | 7 |
| A+T+N- | 0 | 0 | 0 | 0 | 0 |
| A+T+N+ | 17 | 20 | 16 | 4 | 57 |
| Total | 34 | 26 | 31 | 33 |  |

Legend: ATN: AD-related biomarkers system. It is divided into three binary categories based on the nature of the underlying pathophysiology. Biomarkers of fibrillary Aβ deposition (A+) are retention on amyloid (PET or CSF Aβ). Biomarkers of tau pathology (neurofibrillary tangles) (CSF phosphorylated tau (p-tau) and tau PET). Biomarkers of AD-like neurodegeneration or neuronal injury (CSF total tau (t-tau), [^18^F]-fluorodeoxyglucose (FDG)-PET, sMRI) in regions characteristic of AD.

**Table S4.** ANCOVA significant results at baseline.

| Factors | Variables | 95% CI for Mean Difference | | SE | df | t | p | Cohen's d |
| --- | --- | --- | --- | --- | --- | --- | --- | --- |
|  |  | Lower | Upper |  |  |  |  |  |
| ApoE4  (non-Carriers vs. Carriers) | Aβ42 (log10) | -0.144 | -0.023 | 0.031 | 118 | -2.714 | 0.008 | -0.480 |
|  | T-tau (log10) | 0.070 | 0.194 | 0.031 | 118 | 4.211 | <.001 | 0.731 |
|  | P-tau (log10) | 0.096 | 0.239 | 0.036 | 118 | 4.662 | <.001 | 0.809 |
|  | Clux-Normalized | 8.2*10^-4^ | 0.028 | 0.007 | 190 | 2.091 | 0.038 | 0.233 |
|  | Sigma | 0.001 | 0.024 | 0.006 | 190 | 2.178 | 0.031 | 0.242 |
| Disease  Progression  (Converter vs. non-Converter) | Aβ42 (log10) | 0.036 | 0.157 | 0.031 | 118 | 3.147 | 0.002 | 0.561 |
|  | T-tau (log10) | -0.158 | -0.034 | 0.031 | 118 | -3.078 | 0.003 | -0.516 |
|  | P-tau (log10) | -0.184 | -0.042 | 0.036 | 118 | -3.150 | 0.002 | -0.521 |
|  | Clux-Normalized | 0.002 | 0.030 | 0.007 | 190 | 2.248 | 0.026 | 2.248 |
|  | CharPath-Normalized | 3.2*10^-4^ | 0.004 | 9.02*10^-4^ | 190 | 2.330 | 0.021 | 0.289 |
|  | Sigma | 0.001 | 0.025 | 0.006 | 190 | 2.149 | 0.033 | 0.244 |
|  | MMSE | 0.415 | 1.331 | 0.232 | 194 | 3.759 | <.001 | 0.520 |
|  | ADNI-MEM | 0.309 | 0.573 | 0.067 | 194 | 6.573 | <.001 | 0.914 |
|  | L.Hipp | 1.37*10^-4^ | 2.9*10^-4^ | 4.01*10^-4^ | 191 | 5.305 | <.001 | 0.687 |
|  | R.Hipp | 1.3*10^-4^ | 3.03*10^-4^ | 4.32*10^-4^ | 191 | 5.048 | <.001 | 0.655 |
|  | L.E.C | 4.5*10^-5^ | 1.27*10^-4^ | 2.06*10^-4^ | 191 | 4.188 | <.001 | 0.577 |
|  | R.E.C | 4.3*10^-5^ | 1.18*10^-4^ | 1.9*10^-5^ | 191 | 4.219 | <.001 | 0.565 |

Legend: Aβ42: Amyloid-beta 42, P-tau: phosphorylated Tau, T-tau: total Tau, Hipp: hippocampus, E.C: entorhinal cortex, MMSE: Mini-mental state examination, ADNI-MEM: Alzheimer Disease Neuroimaging Initiative composite score for memory, L: left, R: right; log10: logarithmic transformation. SE: the standard error of the estimated mean; df: the degrees of freedom of the model; t: the value of the t-statistic; p: probability values are corrected by Bonferroni. Cohen’s d: size effect, it is not corrected for multiple comparisons.

**Table S5.** ApoE4*disease progression ANCOVA significant interaction effects for T-tau at baseline

|  |
| --- |

| Measure | Group Contrast | Mean difference | 95% CI for Mean Difference | | SE | t | p |
| --- | --- | --- | --- | --- | --- | --- | --- |
|  |  |  | **Lower** | **Upper** |  |  |  |
| T-tau | Converter non-Carrier vs.  non-Converter non-Carriers | -0.273 | -0.532 | -0.015 | 0.100 | -2.738 | 0.041 |
|  | Converter non-Carriers vs.  Converter Carriers | -0.286 | -0.535 | -0.038 | 0.096 | -2.986 | 0.019 |
| P-tau | Converter non-Carriers vs.  non-Converter non-Carriers | 0.189 | 0.062 | 0.315 | 0.049 | 3.891 | < .001 |
|  | non-Converter Carriers vs.  Converter Carriers | -0.280 | -0.416 | -0.145 | 0.052 | -5.383 | < .001 |
|  | non-Converter non-Carriers vs.  non-converter Carriers | -0.244 | -0.373 | -0.114 | 0.050 | -4.905 | < .001 |

Legend: P-tau: phosphorylated tau, T-tau: total Tau. SE: the standard error of the estimated mean; t: value of the t-statistics.

Note. p-values and confidence intervals adjusted for comparing a family of 4 estimates (confidence intervals corrected using the Tukey method). p-values corrected by Bonferroni.

**Table S6.** ApoE4 and disease progression ANCOVA significant results for the Rate of Change (RoC).

| Factors | Measure | 95% CI for Mean Difference | | SE | df | t | p | Cohen's d |
| --- | --- | --- | --- | --- | --- | --- | --- | --- |
|  |  | Lower | Upper |  |  |  |  |  |
| ApoE4  (Carriers vs non-Carriers) | MMSE (rank) | -28.990 | -1.951 | 7.108 | 194 | -2.247 | 0.026 | -0.278 |
|  | R.EC Norm (rank) | -39.043 | -7.659 | 7.955 | 191 | -2.935 | 0.004 | -0.483 |
|  | R.EC Norm (rank) | -38.629 | -7.158 | 7.977 | 191 | -2.870 | 0.005 | -0.487 |
| Disease  Progression  (Converter vs non-Converter) | MMSE (rank) | 42.612 | 70.867 | 7.163 | 194 | 7.921 | <.001 | 1.119 |
|  | ADNI-MEM (rank) | 48.063 | 75.752 | 7.020 | 194 | 8.819 | <.001 | 1.267 |
|  | R.Hipp Norm (rank) | 9.134 | 41.260 | 8.144 | 191 | 3.094 | 0.002 | 0.446 |
|  | L.Hipp Norm (rank) | 8.706 | 40.733 | 8.118 | 191 | 3.045 | 0.003 | 0.437 |
|  | R.EC Norm (rank) | 15.031 | 46.611 | 8.005 | 191 | 3.650 | <.001 | 0.524 |
|  | L.EC Norm (rank) | 10.631 | 42.298 | 8.027 | 191 | 3.297 | 0.001 | 0.473 |
|  | Clux (log10) | 0.023 | 0.307 | 0.072 | 190 | 2.296 | 0.023 | 0.337 |
|  | CharPath (log10) | 0.107 | 0.446 | 0.086 | 190 | 3.221 | 0.002 | 0.473 |
|  | GConnect (log10) | 0.094 | 0.399 | 0.077 | 190 | 3.186 | 0.002 | 0.468 |
|  | Eglobal (log10) | 0.112 | 0.450 | 0.086 | 190 | 3.273 | 0.001 | 0.484 |

Legend: Hipp: hippocampus, E.C: entorhinal cortex, MMSE: Mini-mental state examination, ADNI-MEM: Alzheimer Disease Neuroimaging Initiative composite score for memory, L: left, R: right, log10: logarithm transformation, rank: rank transformation. SE: the standard error of the estimated mean; df: the degrees of freedom of the model; t: the value of the t-statistic; p: probability values are corrected by Bonferroni. Cohen’s d: size effect, it is not corrected for multiple comparisons.

**Table S7.** ApoE4*disease progression significant interaction effects for the variables rate of change

| Measure | Group Contrast | Mean difference | 95% CI for Mean Difference | | SE | t | p |
| --- | --- | --- | --- | --- | --- | --- | --- |
|  |  |  | **Lower** | **Upper** |  |  |  |
| Clux (log10) | Converter non-Carriers vs.  non-Converter non-Carriers | -0.474 | -0.788 | -0.159 | 0.121 | -3.901 | < .001 |
| CharPath-Normalized | Converter Carriers vs.  non-Converter non-Carriers | -0.273 | -0.532 | -0.015 | 0.100 | -2.738 | 0.041 |
|  | Converter non-Carriers vs.  Converter Carriers | -0.286 | -0.535 | -0.038 | 0.096 | -2.986 | 0.019 |

Legend: CharPath-Normalized: Normalized Characteristic path length, Clux: clustering index, log10: logarithm transformation, SE: the standard error of the estimated mean; t: value of the t-statistics.

Note. p-values and confidence intervals adjusted for comparing a family of 4 estimates (confidence intervals corrected using the Tukey method). p-values corrected by Bonferroni.

**Table S8.** ANCOVA significant results for ApoE4 main effect for nodal Normalized Clustering index (Nodal Clux-Normalized). Highlighted in red are the Right supramarginal gyrus (SMG.R) (p-corrected = 0.042, p-uncorrected=0.0005) and Left ACgG anterior cingulate gyrus (ACgG.L) (p-corrected = 0.015, p-uncorrected=0.00061) that show significant differences after FDR correction for multiple comparisons. The rest of the reported p-values are uncorrected p<0.01.

| Structure Names | Structures  Short name | ApoE4-  Mean | ApoE4-  95% Conf. Interval size | ApoE4+  Mean | ApoE4+  95% Conf. Interval | Uncorrected p<0.01 |
| --- | --- | --- | --- | --- | --- | --- |
| Right LiG lingual gyrus | LiG.R | 0.78 | 0.055 | 0.80 | 0.062 | 0.009 |
| Right OFuG occipital fusiform gyrus | OFuG.R | 0.79 | 0.063 | 0.81 | 0.067 | 0.007 |
| Right PoG postcentral gyrus | PoG.R | 0.80 | 0.052 | 0.82 | 0.052 | 0.008 |
| Right PT planum temporale | PT.R | 0.78 | 0.065 | 0.80 | 0.078 | 0.005 |
| Right SMG supramarginal gyrus | SMG.R | 0.82 | 0.059 | 0.85 | 0.058 | 0.0005 |
| Right SOG superior occipital gyrus | SOG.R | 0.82 | 0.066 | 0.84 | 0.073 | 0.0037 |
| Right SPL superior parietal lobule | SPL.R | 0.80 | 0.061 | 0.82 | 0.059 | 0.0086 |
| Right TTG transverse temporal gyrus | TTG.R | 0.81 | 0.082 | 0.85 | 0.083 | 0.0030 |
| Left ACgG anterior cingulate gyrus | ACgG.L | 0.83 | 0.065 | 0.86 | 0.069 | 0.00061 |
| Left AnG angular gyrus | AnG.L | 0.80 | 0.058 | 0.82 | 0.065 | 0.0030 |
| Left LiG lingual gyrus | LiG.L | 0.78 | 0.059 | 0.80 | 0.063 | 0.0071 |
| Left MSFG superior frontal gyrus medial segment | MSFG.L | 0.84 | 0.059 | 0.86 | 0.066 | 0.0091 |
| Left SPL superior parietal lobule | SPL.L | 0.81 | 0.059 | 0.83 | 0.056 | 0.0027 |

**Table S9.** ANCOVA significant results for disease progression main effects for nodal Normalized Clustering index (Nodal Clux-Normalized). The reported p-values are FDR corrected for multiple comparisons.

| Structure Names | Structures  Short name | non-Conv. Mean | non-Conv. 95 % Conf. Interval | Conv. Mean | Conv. 95% Conf. Interval | Corrected  p value  (FDR) |
| --- | --- | --- | --- | --- | --- | --- |
| Right Caudate | Cau.R | 0.91 | 0.066 | 0.88 | 0.061 | 0.015 |
| Right Pallidum | Pal.R | 0.99 | 0.076 | 0.96 | 0.076 | 0.029 |
| Right Putamen | Put.R | 0.89 | 0.054 | 0.87 | 0.059 | 0.012 |
| Right Thalamus Proper | Thal.R | 0.93 | 0.047 | 0.91 | 0.050 | 0.005 |
| Right Ventral DC | VentDC.R | 0.95 | 0.070 | 0.92 | 0.062 | 0.012 |
| Right AIns anterior insula | AIns.R | 0.87 | 0.059 | 0.85 | 0.054 | 0.023 |
| Right AnG angular gyrus | AnG.R | 0.83 | 0.054 | 0.81 | 0.061 | 0.037 |
| Right Calc calcarine cortex | Calc.R | 0.77 | 0.073 | 0.75 | 0.073 | 0.031 |
| Right CO central operculum | CO.R | 0.84 | 0.069 | 0.82 | 0.057 | 0.023 |
| Right Cun cuneus | Cun.R | 0.80 | 0.062 | 0.77 | 0.062 | 0.012 |
| Right FRP frontal pole | FRP.R | 0.90 | 0.066 | 0.87 | 0.070 | 0.024 |
| Right FuG fusiform gyrus | FuG.R | 0.82 | 0.056 | 0.80 | 0.058 | 0.019 |
| Right GRe gyrus rectus | GRe.R | 0.85 | 0.080 | 0.82 | 0.068 | 0.015 |
| Right IOG inferior occipital gyrus | IOG.R | 0.87 | 0.067 | 0.84 | 0.061 | 0.024 |
| Right ITG inferior temporal gyrus | ITG.R | 0.87 | 0.056 | 0.85 | 0.058 | 0.019 |
| Right LiG lingual gyrus | LiG.R | 0.80 | 0.063 | 0.78 | 0.055 | 0.025 |
| Right LOrG lateral orbital gyrus | LOrG.R | 0.87 | 0.070 | 0.84 | 0.067 | 0.031 |
| Right MCgG middle cingulate gyrus | MCgG.R | 0.89 | 0.066 | 0.86 | 0.062 | 0.012 |
| Right MFG middle frontal gyrus | MFG.R | 0.84 | 0.049 | 0.82 | 0.054 | 0.012 |
| Right MOrG medial orbital gyrus | MOrG.R | 0.87 | 0.061 | 0.84 | 0.067 | 0.012 |
| Right MPrG precentral gyrus medial segment | MPrG.R | 0.85 | 0.076 | 0.82 | 0.073 | 0.023 |
| Right MTG middle temporal gyrus | MTG.R | 0.84 | 0.050 | 0.82 | 0.056 | 0.037 |
| Right OpIFG opercular part of the inferior frontal gyrus | OpIFG.R | 0.84 | 0.068 | 0.81 | 0.058 | 0.004 |
| Right OrIFG orbital part of the inferior frontal gyrus | OrIFG.R | 0.86 | 0.072 | 0.82 | 0.081 | 0.015 |
| Right PCgG posterior cingulate gyrus | PCgG.R | 0.89 | 0.069 | 0.86 | 0.062 | 0.012 |
| Right PCu precuneus | PCu.R | 0.83 | 0.059 | 0.80 | 0.062 | 0.019 |
| Right PO parietal operculum | PO.R | 0.85 | 0.080 | 0.82 | 0.073 | 0.022 |
| Right PoG postcentral gyrus | PoG.R | 0.83 | 0.049 | 0.80 | 0.052 | 0.004 |
| Right POrG posterior orbital gyrus | POrG.R | 0.86 | 0.068 | 0.84 | 0.065 | 0.022 |
| Right PP planum polare | PP.R | 0.84 | 0.076 | 0.81 | 0.072 | 0.015 |
| Right PrG precentral gyrus | PrG.R | 0.87 | 0.059 | 0.85 | 0.058 | 0.031 |
| Right SCA subcallosal area | SCA.R | 0.86 | 0.079 | 0.83 | 0.082 | 0.031 |
| Right SFG superior frontal gyrus | SFG.R | 0.88 | 0.049 | 0.85 | 0.054 | 0.005 |
| Right SMC supplementary motor cortex | SMC.R | 0.87 | 0.071 | 0.84 | 0.061 | 0.015 |
| Right SMG supramarginal gyrus | SMG.R | 0.84 | 0.060 | 0.82 | 0.058 | 0.042 |
| Right SOG superior occipital gyrus | SOG.R | 0.85 | 0.070 | 0.82 | 0.068 | 0.012 |
| Right SPL superior parietal lobule | SPL.R | 0.83 | 0.059 | 0.80 | 0.060 | 0.012 |
| Right TMP temporal pole | TMP.R | 0.88 | 0.051 | 0.86 | 0.058 | 0.023 |
| Right TrIFG triangular part of the inferior frontal gyrus | TrIFG.R | 0.86 | 0.062 | 0.83 | 0.067 | 0.012 |
| Left Caudate | Cau.L | 0.90 | 0.067 | 0.88 | 0.066 | 0.040 |
| Left Putamen | Put.L | 0.88 | 0.054 | 0.86 | 0.055 | 0.031 |
| Left Thalamus Proper | Thal.L | 0.94 | 0.053 | 0.92 | 0.050 | 0.012 |
| Left Ventral DC | VentDC.L | 0.93 | 0.066 | 0.90 | 0.067 | 0.012 |
| Left ACgG anterior cingulate gyrus | ACgG.L | 0.86 | 0.067 | 0.83 | 0.068 | 0.015 |
| Left AIns anterior insula | AIns.L | 0.88 | 0.060 | 0.86 | 0.058 | 0.026 |
| Left AOrG anterior orbital gyrus | AOrG.L | 0.85 | 0.073 | 0.82 | 0.068 | 0.039 |
| Left AnG angular gyrus | AnG.L | 0.82 | 0.063 | 0.80 | 0.062 | 0.046 |
| Left Calc calcarine cortex | Calc.L | 0.77 | 0.070 | 0.75 | 0.069 | 0.025 |
| Left CO central operculum | CO.L | 0.85 | 0.069 | 0.82 | 0.063 | 0.012 |
| Left FRP frontal pole | FRP.L | 0.90 | 0.071 | 0.87 | 0.066 | 0.012 |
| Left FuG fusiform gyrus | FuG.L | 0.81 | 0.054 | 0.79 | 0.059 | 0.025 |
| Left GRe gyrus rectus | GRe.L | 0.83 | 0.082 | 0.80 | 0.064 | 0.012 |
| Left ITG inferior temporal gyrus | ITG.L | 0.87 | 0.053 | 0.85 | 0.054 | 0.022 |
| Left LOrG lateral orbital gyrus | LOrG.L | 0.87 | 0.076 | 0.85 | 0.067 | 0.022 |
| Left MCgG middle cingulate gyrus | MCgG.L | 0.86 | 0.062 | 0.83 | 0.069 | 0.013 |
| Left MFG middle frontal gyrus | MFG.L | 0.85 | 0.053 | 0.82 | 0.053 | 0.005 |
| Left MOrG medial orbital gyrus | MOrG.L | 0.88 | 0.069 | 0.85 | 0.066 | 0.015 |
| Left MSFG superior frontal gyrus medial segment | MSFG.L | 0.86 | 0.062 | 0.84 | 0.064 | 0.025 |
| Left MTG middle temporal gyrus | MTG.L | 0.84 | 0.055 | 0.82 | 0.055 | 0.023 |
| Left OCP occipital pole | OCP.L | 0.85 | 0.069 | 0.82 | 0.072 | 0.023 |
| Left OpIFG opercular part of the inferior frontal gyrus | OpIFG.L | 0.83 | 0.064 | 0.81 | 0.072 | 0.041 |
| Left PCgG posterior cingulate gyrus | PCgG.L | 0.87 | 0.065 | 0.84 | 0.061 | 0.018 |
| Left PCu precuneus | PCu.L | 0.81 | 0.056 | 0.80 | 0.059 | 0.031 |
| Left PO parietal operculum | PO.L | 0.84 | 0.072 | 0.81 | 0.070 | 0.025 |
| Left PoG postcentral gyrus | PoG.L | 0.83 | 0.052 | 0.81 | 0.056 | 0.019 |
| Left POrG posterior orbital gyrus | POrG.L | 0.85 | 0.066 | 0.82 | 0.063 | 0.021 |
| Left PP planum polare | PP.L | 0.82 | 0.069 | 0.80 | 0.068 | 0.031 |
| Left PrG precentral gyrus | PrG.L | 0.87 | 0.061 | 0.84 | 0.052 | 0.012 |
| Left SFG superior frontal gyrus | SFG.L | 0.88 | 0.049 | 0.85 | 0.056 | 0.012 |
| Left SMG supramarginal gyrus | SMG.L | 0.85 | 0.060 | 0.83 | 0.058 | 0.019 |
| Left SOG superior occipital gyrus | SOG.L | 0.83 | 0.075 | 0.80 | 0.073 | 0.019 |
| Left SPL superior parietal lobule | SPL.L | 0.83 | 0.056 | 0.81 | 0.059 | 0.029 |
| Left STG superior temporal gyrus | STG.L | 0.83 | 0.058 | 0.80 | 0.066 | 0.015 |
| Left TMP temporal pole | TMP.L | 0.88 | 0.054 | 0.85 | 0.055 | 0.016 |
| Left TrIFG triangular part of the inferior frontal gyrus | TrIFG.L | 0.87 | 0.069 | 0.85 | 0.068 | 0.034 |

**Table S10**. Correlation between network topological attributes and MMSE taking all data sample and dividing groups into Converts and non-Converters and APOE4+, APOE4-. The significant values are represented in red.

| Topological Variables | General Corr. | p - General Corr. | Conv. Corr. | p –  Conv. Corr. | non-Conv. Corr. | p – non-Conv. Corr. | APOE4+ Corr. | p –  APOE4+ Corr. | APOE4-Corr. | p - APOE4-Corr. |
| --- | --- | --- | --- | --- | --- | --- | --- | --- | --- | --- |
| Clux Normalized | 0.12 | 0.094 | 0.007 | 0.94 | 0.095 | 0.35 | 0.09 | 0.36 | 0.15 | 0.12 |
| Clux | 0.12 | 0.070 | -0.05 | 0.62 | 0.18 | 0.064 | 0.04 | 0.69 | 0.21 | 0.03 |
| CharPathL Normalized | 0.14 | 0.038 | 0.105 | 0.30 | 0.068 | 0.50 | 0.15 | 0.12 | 0.14 | 0.16 |
| CharPathL | 0.014 | 0.84 | 0.095 | 0.35 | -0.11 | 0.25 | 0.05 | 0.57 | -0.02 | 0.82 |
| Global Connectivity | 0.081 | 0.25 | -0.086 | 0.40 | 0.203 | 0.046 | 0.005 | 0.96 | 0.15 | 0.13 |
| Eglobal | 0.042 | 0.55 | -0.092 | 0.37 | 0.17 | 0.09 | -0.02 | 0.83 | 0.10 | 0.32 |

**Table S11**. Correlation between network topological attributes and ADNI-MEM taking all data sample and dividing groups into Converts and non-Converters and APOE4+, APOE4-. The significant values are represented in red.

| Topological Variables | General Corr. | p - General Corr. | Conv. Corr. | p – Conv. Corr. | non-Conv. Corr. | p - non-Conv. Corr. | APOE4+ Corr. | p – APOE4+ Corr. | APOE4-Corr. | p - APOE4-Corr. |
| --- | --- | --- | --- | --- | --- | --- | --- | --- | --- | --- |
| Clux  Normalized | **0.206** | **0.003** | 0.09 | 0.37 | 0.071 | 0.48 | **0.22** | **0.030** | **0.234** | **0.021** |
| Clux | **0.26** | **2*10^-4^** | 0.12 | 0.22 | **0.25** | **0.014** | 0.13 | 0.176 | **0.388** | **8.97*10^-5^** |
| CharPathL Normalized | **0.246** | **5*10^-4^** | 0.17 | 0.08 | 0.072 | 0.48 | **0.29** | **0.003** | **0.232** | **0.022** |
| CharPathL | 0.019 | 0.785 | 0.07 | 0.49 | -0.145 | 0.15 | 0.12 | 0.243 | -0.061 | 0.55 |
| Global  Connectivity | **0.162** | **0.023** | 0.040 | 0.69 | **0.24** | **0.016** | 0.03 | 0.705 | **0.287** | **0.004** |
| Eglobal | 0.087 | 0.22 | -0.007 | 0.93 | 0.20 | 0.040 | -0.02 | 0.785 | 0.198 | 0.051 |

**Table S12**. Correlation between network topological attributes and CSF measure Aβ42 taking all data sample and dividing groups into Converts and non-Converters and APOE4+, APOE4-. The significant values are represented in red.

| Topological Variables | General Corr. | p - General Corr. | Conv. Corr. | p – Conv. Corr. | non-Conv. Corr. | p - non-Conv. Corr. | APOE4+ Corr. | p – APOE4+ Corr. | APOE4-Corr. | p - APOE4-Corr. |
| --- | --- | --- | --- | --- | --- | --- | --- | --- | --- | --- |
| Clux  Normalized | 0.08 | 0.36 | 0.035 | 0.78 | -0.053 | 0.69 | 0.25 | 0.06 | 0.006 | 0.95 |
| Clux | 0.07 | 0.41 | 0.053 | 0.68 | -0.031 | 0.81 | -0.03 | 0.81 | 0.15 | 0.21 |
| CharPathL Normalized | 0.10 | 0.27 | 0.033 | 0.80 | -0.009 | 0.94 | 0.28 | 0.039 | -0.03 | 0.75 |
| CharPathL | 0.006 | 0.94 | -0.02 | 0.84 | -0.014 | 0.91 | 0.22 | 0.09 | -0.17 | 0.18 |
| Global  Connectivity | 0.069 | 0.45 | 0.065 | 0.61 | 0.012 | 0.92 | -0.11 | 0.41 | 0.21 | 0.08 |
| Eglobal | 0.038 | 0.67 | 0.049 | 0.70 | 0.013 | 0.91 | -0.16 | 0.22 | 0.20 | 0.10 |

**Table S13**. Correlation between network topological attributes and CSF measure Tau taking all data sample and dividing groups into Converts and non-Converters and APOE4+, APOE4-. The significant values are represented in red.

| Topological Variables | General Corr. | p - General Corr. | Conv. Corr. | p – Conv. Corr. | non-Conv. Corr. | p –  non-Conv. Corr. | APOE4+ Corr. | p – APOE4+ Corr. | APOE4-Corr. | p - APOE4-Corr. |
| --- | --- | --- | --- | --- | --- | --- | --- | --- | --- | --- |
| Clux  Normalized | -0.06 | 0.51 | -0.08 | 0.52 | 0.12 | 0.37 | -0.11 | 0.39 | -0.14 | 0.25 |
| Clux | -0.005 | 0.95 | 0.05 | 0.66 | 0.02 | 0.87 | -0.02 | 0.88 | -0.037 | 0.76 |
| CharPathL Normalized | -0.05 | 0.56 | -0.06 | 0.63 | 0.15 | 0.26 | -0.05 | 0.72 | -0.10 | 0.40 |
| CharPathL | -0.03 | 0.72 | -0.08 | 0.54 | 0.10 | 0.42 | -0.005 | 0.96 | -0.06 | 0.60 |
| Global  Connectivity | 0.005 | 0.95 | 0.07 | 0.57 | -0.04 | 0.76 | -0.029 | 0.83 | 0.01 | 0.93 |
| Eglobal | 0.017 | 0.84 | 0.07 | 0.54 | -0.07 | 0.60 | -0.015 | 0.91 | 0.03 | 0.78 |

**Table S14**. Correlation between network topological attributes and CSF measure pTau taking all data sample and dividing groups into Converts and non-Converters and APOE4+, APOE4-. The significant values are represented in red.

| Topological Variables | General Corr. | p - General Corr. | Conv. Corr. | p – Conv. Corr. | non-Conv. Corr. | p – non-Conv. Corr. | APOE4+ Corr. | p – APOE4+ Corr. | APOE4-Corr. | p - APOE4-Corr. |
| --- | --- | --- | --- | --- | --- | --- | --- | --- | --- | --- |
| Clux  Normalized | -0.04 | 0.63 | -0.08 | 0.50 | 0.16 | 0.22 | -0.13 | 0.34 | -0.11 | 0.361 |
| Clux | -0.01 | 0.89 | 0.03 | 0.81 | 0.027 | 0.83 | -0.02 | 0.88 | -0.045 | 0.72 |
| CharPathL Normalized | -0.04 | 0.61 | -0.06 | 0.60 | 0.17 | 0.20 | -0.07 | 0.58 | -0.077 | 0.54 |
| CharPathL | -0.02 | 0.82 | -0.06 | 0.61 | 0.12 | 0.37 | -0.026 | 0.85 | -0.034 | 0.78 |
| Global  Connectivity | -0.007 | 0.93 | 0.04 | 0.71 | -0.04 | 0.73 | -0.02 | 0.88 | -0.012 | 0.92 |
| Eglobal | 0.004 | 0.96 | 0.05 | 0.65 | -0.07 | 0.56 | -0.0009 | 0.99 | 0.0076 | 0.95 |

**Table S15**. Statistical differences APOE4+ vs. APOE4- and Converters (Conv) vs. non-Converters (non-Conv) in the linear correlation between topological network attributes and MMSE. The topological network attributes are: Clustering index normalized (Clux Normalized), Clustering index (Clux), Characteristic path length Normalized (CharPathL Normalized), Global connectivity, and global efficiency (Eglobal). The significant values are represented in red.

| Topological Variables | Conv. Corr. | non-Conv. Corr. | Z-Stats (Conv. vs. non-Conv.) | p  (Conv. vs. non-Conv.) | APOE4+ Corr. | APOE4- Corr. | Z-Stats  (APOE4+ vs. APOE4-) | p  (APOE4+ vs. APOE4-) |
| --- | --- | --- | --- | --- | --- | --- | --- | --- |
| Clux  Normalized | 0.095 | 0.007 | 0.607 | 0.54 | 0.094 | 0.15 | -0.457 | 0.64 |
| Clux | 0.189 | -0.05 | 1.67 | 0.09 | 0.040 | 0.21 | -1.243 | 0.21 |
| CharPathL  Normalized | 0.068 | 0.105 | -0.25 | 0.79 | 0.15 | 0.14 | 0.101 | 0.91 |
| CharPathL | -0.11 | 0.095 | -1.46 | 0.14 | 0.05 | -0.02 | 0.559 | 0.57 |
| Global  Connectivity | **0.20** | **-0.08** | **2.01** | **0.04** | 0.005 | 0.15 | -1.033 | 0.30 |
| Eglobal | 0.17 | -0.09 | 1.84 | 0.065 | -0.022 | 0.10 | -0.856 | 0.39 |

Legend: **Conv. Corr** : Converters group partial correlation coefficient; **non-Conv. Corr**: non-Converters group partial correlation coefficient; **Z-Stats (Conv. vs. non-Conv.)**: Z-stats Converters vs. non-Converters correlation coefficients; **p (Conv. vs. non-Conv.)**: p-value of the Z-stats Converters vs. non-Converters; **APOE4+ Corr.** : APOE4+ group partial correlation coefficient; **APOE4- Corr.** : APOE4- group partial correlation coefficient; **Z-Stats (APOE4+ vs. APOE4-)** Z-stats APOE4+ vs. APOE4- correlation coefficients; **p (APOE4+ vs. APOE4-)** p-value of the Z-Stats (APOE4+ vs. APOE4-).

**Table S16**. Statistical differences APOE4+ vs. APOE4- and Converters (Conv) vs. non-Converters (non-Conv) in the linear correlation between topological network attributes and ADNI-MEM. The topological network attributes are: Clustering index normalized (Clux Normalized), Clustering index (Clux), Characteristic path length Normalized (CharPathL Normalized), Global connectivity, and global efficiency (Eglobal). The significant values are represented in red.

| Topological Variables | Conv. Corr. | non-Conv. Corr. | Z-Stats  (Conv. vs. non-Conv.) | p  (Conv. vs. non-Conv.) | APOE4+ Corr. | APOE4- Corr. | Z-Stats (APOE4+ vs. APOE4-) | p  (APOE4+ vs. APOE4-) |
| --- | --- | --- | --- | --- | --- | --- | --- | --- |
| Clux  Normalized | 0.071 | 0.09 | -0.143 | 0.88 | 0.22 | 0.23 | -0.08 | 0.93 |
| Clux | 0.25 | 0.12 | 0.88 | 0.37 | 0.14 | 0.39 | -1.85 | 0.063 |
| CharPathL Normalized | 0.072 | 0.17 | -0.71 | 0.47 | 0.29 | 0.23 | 0.48 | 0.63 |
| CharPathL | -0.14 | 0.07 | -1.49 | 0.13 | 0.12 | -0.06 | 1.25 | 0.20 |
| Global  Connectivity | 0.24 | 0.04 | 1.43 | 0.15 | 0.039 | 0.29 | -1.76 | 0.078 |
| Eglobal | 0.20 | -0.007 | 1.51 | 0.12 | -0.028 | 0.199 | -1.58 | 0.11 |

Legend: **Conv. Corr** : Converters group partial correlation coefficient; **non-Conv. Corr**: non-Converters group partial correlation coefficient; **Z-Stats (Conv. vs. non-Conv.)**: Z-stats Converters vs. non-Converters correlation coefficients; **p (Conv. vs. non-Conv.)**: p-value of the Z-stats Converters vs. non-Converters; **APOE4+ Corr.** : APOE4+ group partial correlation coefficient; **APOE4- Corr.** : APOE4- group partial correlation coefficient; **Z-Stats (APOE4+ vs. APOE4-)** Z-stats APOE4+ vs. APOE4- correlation coefficients; **p (APOE4+ vs. APOE4-)** p-value of the Z-Stats (APOE4+ vs. APOE4-).

**Table S17**. Statistical differences APOE4+ vs. APOE4- and Converters (Conv) vs. non-Converters (non-Conv) in the linear correlation between topological network attributes and CSF measure Aβ42. The topological network attributes are: Clustering index normalized (Clux Normalized), Clustering index (Clux), Characteristic path length Normalized (CharPathL Normalized), Global connectivity, and global efficiency (Eglobal). The significant values are represented in red.

| Topological  Variables | Conv. Corr. | non-Conv. Corr. | Z-Stats (Conv. vs. non-Conv.) | p  (Conv. vs. non-Conv.) | APOE4+ Corr. | APOE4- Corr. | Z-Stats (APOE4+ vs. APOE4-) | p  (APOE4+ vs. APOE4-) |
| --- | --- | --- | --- | --- | --- | --- | --- | --- |
| Clux  Normalized | -0.05 | 0.035 | -0.47 | 0.63 | 0.25 | 0.006 | 1.34 | 0.17 |
| Clux | -0.03 | 0.053 | -0.45 | 0.65 | -0.03 | 0.158 | -1.02 | 0.30 |
| CharPathL Normalized | -0.009 | 0.033 | -0.22 | 0.81 | 0.28 | -0.039 | 1.76 | 0.078 |
| CharPathL | -0.014 | -0.02 | 0.057 | 0.95 | **0.229** | **-0.171** | **2.15** | **0.03** |
| Global  Connectivity | 0.012 | 0.06 | -0.28 | 0.77 | -0.11 | 0.216 | -1.77 | 0.07 |
| Eglobal | 0.013 | 0.04 | -0.18 | 0.85 | **-0.16** | **0.20** | **-2.00** | **0.044** |

Legend: **Conv. Corr** : Converters group partial correlation coefficient; **non-Conv. Corr**: non-Converters group partial correlation coefficient; **Z-Stats (Conv. vs. non-Conv.)**: Z-stats Converters vs. non-Converters correlation coefficients; **p (Conv. vs. non-Conv.)**: p-value of the Z-stats Converters vs. non-Converters; **APOE4+ Corr.** : APOE4+ group partial correlation coefficient; **APOE4- Corr.** : APOE4- group partial correlation coefficient; **Z-Stats (APOE4+ vs. APOE4-)** Z-stats APOE4+ vs. APOE4- correlation coefficients; **p (APOE4+ vs. APOE4-)** p-value of the Z-Stats (APOE4+ vs. APOE4-).

**Table S18**. Statistical differences APOE4+ vs. APOE4- and Converters (Conv) vs. non-Converters (non-Conv) in the linear correlation between topological network attributes and CSF measure T-tau. The topological network attributes are: Clustering index normalized (Clux Normalized), Clustering index (Clux), Characteristic path length Normalized (CharPathL Normalized), Global connectivity, and global efficiency (Eglobal). The significant values are represented in red.

| Topological Variables | Conv.  Corr. | non-Conv. Corr. | Z-Stats (Conv. vs. non-Conv.) | p  (Conv. vs. non-Conv.) | APOE4+ Corr. | APOE4- Corr. | Z-Stats  (APOE4+ vs. APOE4-) | p  (APOE4+ vs. APOE4-) |
| --- | --- | --- | --- | --- | --- | --- | --- | --- |
| Clux  Normalized | 0.12 | -0.08 | 1.09 | 0.27 | -0.11 | -0.14 | 0.14 | 0.88 |
| Clux | 0.02 | 0.05 | -0.18 | 0.85 | -0.02 | -0.037 | 0.09 | 0.92 |
| CharPathL  Normalized | 0.15 | -0.06 | 1.15 | 0.24 | -0.05 | -0.10 | 0.30 | 0.75 |
| CharPathL | 0.10 | -0.08 | 1.006 | 0.31 | -0.005 | -0.066 | 0.32 | 0.74 |
| Global  Connectivity | -0.041 | 0.07 | -0.61 | 0.53 | -0.029 | 0.010 | -0.21 | 0.83 |
| Eglobal | -0.07 | 0.07 | -0.79 | 0.42 | -0.015 | 0.035 | -0.27 | 0.78 |

Legend: **Conv. Corr** : Converters group partial correlation coefficient; **non-Conv. Corr**: non-Converters group partial correlation coefficient; **Z-Stats (Conv. vs. non-Conv.)**: Z-stats Converters vs. non-Converters correlation coefficients; **p (Conv. vs. non-Conv.)**: p-value of the Z-stats Converters vs. non-Converters; **APOE4+ Corr.** : APOE4+ group partial correlation coefficient; **APOE4- Corr.** : APOE4- group partial correlation coefficient; **Z-Stats (APOE4+ vs. APOE4-)** Z-stats APOE4+ vs. APOE4- correlation coefficients; **p (APOE4+ vs. APOE4-)** p-value of the Z-Stats (APOE4+ vs. APOE4-).

**Table S19**. Statistical differences APOE4+ vs. APOE4- and Converters (Conv) vs. non-Converters (non-Conv) in the linear correlation between topological network attributes and CSF measure P-tau. The topological network attributes are: Clustering index normalized (Clux Normalized), Clustering index (Clux), Characteristic path length Normalized (CharPathL Normalized), Global connectivity, and global efficiency (Eglobal). The significant values are represented in red.

| Topological  Variables | Conv.  Corr. | non-Conv. Corr. | Z-Stats (Conv. vs. non-Conv.) | p  (Conv. vs. non-Conv.) | APOE4+ Corr. | APOE4- Corr. | Z-Stats (APOE4+ vs. APOE4-) | p  (APOE4+ vs. APOE4-) |
| --- | --- | --- | --- | --- | --- | --- | --- | --- |
| Clux  Normalized | 0.16 | -0.087 | 1.34 | 0.178 | -0.13 | -0.11 | -0.07 | 0.93 |
| Clux | 0.027 | 0.030 | -0.01 | 0.99 | -0.02 | -0.04 | 0.13 | 0.89 |
| CharPathL  Normalized | 0.17 | -0.067 | 1.29 | 0.19 | -0.07 | -0.07 | 0.004 | 0.99 |
| CharPathL | 0.12 | -0.066 | 1.003 | 0.31 | -0.02 | -0.03 | 0.04 | 0.96 |
| Global  Connectivity | -0.04 | 0.048 | -0.50 | 0.61 | -0.02 | -0.01 | -0.04 | 0.96 |
| Eglobal | -0.07 | 0.058 | -0.73 | 0.46 | -0.00091 | 0.007 | -0.04 | 0.96 |

Legend: **Conv. Corr** : Converters group partial correlation coefficient; **non-Conv. Corr**: non-Converters group partial correlation coefficient; **Z-Stats (Conv. vs. non-Conv.)**: Z-stats Converters vs. non-Converters correlation coefficients; **p (Conv. vs. non-Conv.)**: p-value of the Z-stats Converters vs. non-Converters; **APOE4+ Corr.** : APOE4+ group partial correlation coefficient; **APOE4- Corr.** : APOE4- group partial correlation coefficient; **Z-Stats (APOE4+ vs. APOE4-)** Z-stats APOE4+ vs. APOE4- correlation coefficients; **p (APOE4+ vs. APOE4-)** p-value of the Z-Stats (APOE4+ vs. APOE4-).

**Experimental Groups selection procedure**

Our study focused on the late MCI (LMCI) present in ADNI1, ADNIGO, and ADNI2 phases. One reason to aim the attention at late MCI is that LMCI subjects have more probability (at higher risk) of converting to AD during the ADNI study. ADNIGO by design provides only early MCI (EMCI); therefore, no new LMCI subjects were included from this ADNI phase. The total number of LMCIs was 566; two didn’t have the APGEN information in the ‘APOERES.csv’ table. We excluded the e4-e4 = 73 and e2 = 43. So, our study’s total number of candidate subjects was N = 564 – 116 = 448 subjects.

The following table shows the information concerning the groups subdivided following the disease progression and presence of the e4 allele (APOE4+, APOE4-) variables.

**Table S20.** Information concerning the groups subdivided following the disease progression and presence of the e4 allele (APOE4+, APOE4-) variables.

|  | LMCI non-Converters | | LMCI Converters | |
| --- | --- | --- | --- | --- |
|  | Carriers (APOE4+) | Non-Carriers (APO4-) | Carriers (APOE4+) | Non-Carriers (APO4-) |
| Number of subjects | 80 | 131 | 139 | 98 |

The smallest group was the Carriers (APOE4+) non-converters with 80 subjects. We excluded some subjects at different stages of our analysis from this pool (and the rest of the cohort) due to: change of strength field between visits, MRI scanner hardware changes reported as significant changes (i.e., head coil, MRI equipment) between time points. Also, we excluded subjects with low-quality gray matter segmentation in one or two time points during the networks computation step.

We finalized with 54 subjects out of 80 in this group. The other three groups were created to match as much as possible this group (APOE4+ non-converters) in terms of age and gender. Finally, a few subjects from this group were separated to achieve a better match across groups, yielding 50 subjects.

**Influence of traditional markers for AD on the relationship between APOE4 and disease progression with Network measures.**

The significant values are represented in red.

**Table S21.** Influence of Aβ42 on the relationship between APOE4 and disease progression with Network measures.

| Factors | Variables | F | p | ω2 |
| --- | --- | --- | --- | --- |
|  |  |  |  |  |
| ApoE4  (non-Carriers vs. Carriers) | Sigma | 2.941 | 0.089 | 0.01 |
|  | CharPath-Normalized | 0.119 | 0.730 | 0.000 |
|  | Clux-Normalized | 2.272 | 0.135 | 0.006 |
| Disease  Progression  (Converter vs. non-Converter) | Sigma | 0.703 | 0.404 | 0.000 |
|  | CharPath-Normalized | 1.070 | 0.303 | 4.25^-4^ |
|  | Clux-Normalized | 0.806 | 0.371 | 0.000 |
| ApoE4* Disease  Progression | Sigma | 2.273 | 0.134 | 0.006 |
|  | CharPath-Normalized |  |  |  |
|  | Clux-Normalized | 1.767 | 0.186 | 0.004 |

**Table S22.** Influence of T-tau on the relationship between APOE4 and disease progression with Network measures.

| Factors | Variables | F | p | ω2 |
| --- | --- | --- | --- | --- |
|  |  |  |  |  |
| ApoE4  (non-Carriers vs. Carriers) | Sigma | 3.740 | 0.056 | 0.013 |
|  | CharPath-Normalized | 0.125 | 0.724 | 0.000 |
|  | Clux-Normalized | 2.854 | 0.094 | 0.009 |
| Disease  Progression  (Converter vs. non-Converter) | Sigma | 0.460 | 0.499 | 0.000 |
|  | CharPath-Normalized | 1.044 | 0.309 | 2.655^-4^ |
|  | Clux-Normalized | 0.584 | 0.446 | 0.000 |
| ApoE4* Disease  Progression | Sigma | 1.719 | 0.192 | 0.004 |
|  | CharPath-Normalized | 0.052 | 0.820 | 0.000 |
|  | Clux-Normalized | 1.346 | 0.248 | 0.002 |

**Table S23.** Influence of P-tau on the relationship between APOE4 and disease progression with Network measures.

| Factors | Variables | F | p | ω2 |
| --- | --- | --- | --- | --- |
|  |  |  |  |  |
| ApoE4  (non-Carriers vs. Carriers) | Sigma | 3.700 | 0.057 | 0.013 |
|  | CharPath-Normalized | 0.120 | 0.729 | 0.000 |
|  | Clux-Normalized | 2.824 | 0.096 | 0.009 |
| Disease  Progression  (Converter vs. non-Converter) | Sigma | 0.494 | 0.483 | 0.000 |
|  | CharPath-Normalized | 1.055 | 0.307 | 3.312^-4^ |
|  | Clux-Normalized | 0.618 | 0.433 | 0.000 |
| ApoE4* Disease  Progression | Sigma | 1.774 | 0.186 | 0.004 |
|  | CharPath-Normalized | 0.053 | 0.819 | 0.000 |
|  | Clux-Normalized | 1.387 | 0.241 | 0.002 |

**Table S24.** Influence of Right Hippocampus Normalized Volume on the relationship between APOE4 and disease progression with Network measures.

| Factors | Variables | F | p | ω2 |
| --- | --- | --- | --- | --- |
|  |  |  |  |  |
| ApoE4  (non-Carriers vs. Carriers) | Sigma | 10.22 | 0.002* | 0.030 |
|  | CharPath-Normalized | 5.055 | 0.026* | 0.015 |
|  | Clux-Normalized | 9.738 | 0.002* | 0.029 |
| Disease  Progression  (Converter vs. non-Converter) | Sigma | 0.024 | 0.877 | 0.000 |
|  | CharPath-Normalized | 0.219 | 0.640 | 0.000 |
|  | Clux-Normalized | 0.056 | 0.814 | 0.000 |
| ApoE4* Disease  Progression | Sigma | 0.055 | 0.772 | 0.000 |
|  | CharPath-Normalized | 0.546 | 0.461 | 0.000 |
|  | Clux-Normalized | 0.017 | 0.895 | 0.000 |

**Table S25.** Influence of Right Hippocampus Normalized Volume on the relationship between APOE4 and disease progression with Network measures.

| Factors | Variables | F | p | ω2 |
| --- | --- | --- | --- | --- |
|  |  |  |  |  |
| ApoE4  (non-Carriers vs. Carriers) | Sigma | 9.642 | 0.002* | 0.028 |
|  | CharPath-Normalized | 5.039 | 0.026* | 0.015 |
|  | Clux-Normalized | 9.224 | 0.003* | 0.027 |
| Disease  Progression  (Converter vs. non-Converter) | Sigma | 0.058 | 0.811 | 0.000 |
|  | CharPath-Normalized | 0.207 | 0.649 | 0.000 |
|  | Clux-Normalized | 0.094 | 0.760 | 0.000 |
| ApoE4* Disease  Progression | Sigma | 0.364 | 0.547 | 0.000 |
|  | CharPath-Normalized | 0.210 | 0.647 | 0.000 |
|  | Clux-Normalized | 0.202 | 0.654 | 0.000 |

**Reliability evaluation of the *SSGMNets* topological network properties**

To study the reliability of the *SSGMNets* topological properties, we used the KKI (Kennedy Krieger Institute—Multi-Modal MRI Reproducibility Resource) data available at <https://www.nitrc.org/projects/multimodal/>.

In summary, this dataset comprises 21 healthy volunteers with no history of neurological conditions (10 F, 22–61 years old). The data were acquired using a 3T MRI scanner (Achieva, Philips Healthcare, Best, The Netherlands) with body coil excitation and an eight-channel phased-array SENSitivity Encoding (SENSE) head-coil for the reception. All scans were completed during a 2-week interval. The resulting data set consisted of 42 "1-h" sessions of 21 individuals. MP-RAGE T1-weighted scans were acquired with a 3D inversion recovery sequence: (TR/TE/TI = 6.7/3.1/842 ms) with a 1.0 ×1.0 × 1.2mm3 resolution over a field of view of 240 × 204 × 256mm acquired in the sagittal plane. The SENSE acceleration factor was 2 in the right-left direction. Multi-shot fast gradient echo (TFE factor = 240) was used with a 3-s shot interval and the turbo direction being in the slice direction (right–left). The flip angle was 8°. No fat saturation was employed.

References:

Landis, J. R. & Koch, G. G. The measurement of observer agreement for categorical data. Biometrics 33, 159–174 (1977).

Bennett. A. Landman, Alan J. Huang, Aliya Gifford, Deepti S. Vikram, Issel Anne L. Lim, Jonathan A.D. Farrell, John A. Bogovic, Jun Hua, Min Chen, Samson Jarso, Seth A. Smith, Suresh Joel, Susumu Mori, James J. Pekar, Peter B. Barker, Jerry L. Prince, and Peter C.M. van Zijl. "Multi-Parametric Neuroimaging Reproducibility: A 3T Resource Study", NeuroImage. (2010) NIHMS/PMC:252138 doi:10.1016/j.neuroimage.2010.11.047

Pizzagalli, Fabrizio, et al. "The reliability and heritability of cortical folds and their genetic correlations across hemispheres." Communications biology 3.1 (2020): 1-12.

**Table S26**. Reproducibility analysis for the global network properties.

| Network Properties | ICC | FValue | df1 | df2 | pVal | 95% Confidence Interval |
| --- | --- | --- | --- | --- | --- | --- |
| Clux | 0.84 | 11.93 | 20 | 21 | 2.1*10^-7^ | (0.66, 0.93) |
| CharPathL | 0.72 | 6.39 | 20 | 21 | 4.2*10^-5^ | (0.45, 0.87) |
| Eglobal | 0.78 | 8.27 | 20 | 21 | 5.2*10^-6^ | (0.54, 0.90) |
| Global Connectivity | 0.83 | 10.73 | 20 | 21 | 5.4*10^-7^ | (0.63, 0.92) |
| Number of Nodes | 0.99 | 1704 | 20 | 21 | 1*10^-200^ | (0.99, 0.991) |
| Clux normalized | 0.82 | 10.29 | 20 | 21 | 7.9*10^-7^ | (0.61, 0.92) |
| CharPathL normalized | 0.80 | 9.0 | 20 | 21 | 2.5*10^-6^ | (0.57, 0.91) |
| sigma | 0.80 | 9.12 | 20 | 21 | 2.2*10^-6^ | (0.58, 0.91) |

**Table S27**. Reproducibility analysis for the regional clustering index normalized. In red the anatomical structures with APOE4 and disease progression significant effects.

| Structure Name | ICC | FValue | df1 | df2 | pVal | 95% Confidence Interval |
| --- | --- | --- | --- | --- | --- | --- |
| Accum.R | 0.29 | 1.84 | 20 | 21 | 0.086 | (-0.1,0.63) |
| Amyg.R | 0.54 | 3.39 | 20 | 21 | 0.003 | (0.16,0.78) |
| Cau.R | 0.58 | 3.82 | 20 | 21 | 0.001 | (0.22,0.80) |
| Hip.R | 0.75 | 7.11 | 20 | 21 | 1.81*10^-5^ | (0.49,0.89) |
| Pal.R | 0.52 | 3.24 | 20 | 21 | 0.005 | (0.14,0.77) |
| Put.R | 0.65 | 4.77 | 20 | 21 | 0.0003 | (0.32,0.84) |
| Thal.R | 0.50 | 3.00 | 20 | 21 | 0.007 | (0.10,0.76) |
| VentDC.R | 0.30 | 1.86 | 20 | 21 | 0.083 | (-0.1,0.63) |
| ACgG.R | 0.65 | 4.78 | 20 | 21 | 0.0003 | (0.32,0.84) |
| AIns.R | 0.25 | 1.67 | 20 | 21 | 0.12 | (-0.1,0.60) |
| AOrG.R | 0.53 | 3.31 | 20 | 21 | 0.004 | (0.15,0.78) |
| AnG.R | 0.51 | 3.09 | 20 | 21 | 0.006 | (0.12,0.76) |
| Calc.R | 0.49 | 2.96 | 20 | 21 | 0.008 | (0.10,0.75) |
| CO.R | 0.25 | 1.67 | 20 | 21 | 0.12 | (-0.1,0.60) |
| Cun.R | 0.26 | 1.70 | 20 | 21 | 0.11 | (-0.1,0.61) |
| Ent.R | 0.65 | 4.75 | 20 | 21 | 0.0004 | (0.32,0.84) |
| FO.R | 0.58 | 3.85 | 20 | 21 | 0.001 | (0.22,0.80) |
| FRP.R | 0.62 | 4.39 | 20 | 21 | 0.0006 | (0.28,0.82) |
| FuG.R | 0.67 | 5.19 | 20 | 21 | 0.0002 | (0.36,0.85) |
| GRe.R | 0.61 | 4.15 | 20 | 21 | 0.001 | (0.26,0.82) |
| IOG.R | 0.69 | 5.54 | 20 | 21 | 0.0001 | (0.39,0.86) |
| ITG.R | 0.58 | 3.86 | 20 | 21 | 0.001 | (0.22,0.80) |
| LiG.R | 0.77 | 7.90 | 20 | 21 | 7.61*10^-6^ | (0.53,0.90) |
| LOrG.R | 0.55 | 3.48 | 20 | 21 | 0.003 | (0.17,0.79) |
| MCgG.R | 0.85 | 12.3 | 20 | 21 | 1.56*10^-7^ | (0.67,0.93) |
| MFC.R | 0.64 | 4.57 | 20 | 21 | 0.0005 | (0.30,0.83) |
| MFG.R | 0.78 | 8.28 | 20 | 21 | 5.162*10^-6^ | (0.54,0.90) |
| MOG.R | 0.23 | 1.61 | 20 | 21 | 0.14 | (-0.1,0.59) |
| MOrG.R | 0.70 | 5.72 | 20 | 21 | 0.0001 | (0.40,0.86) |
| MPoG.R | 0.45 | 2.66 | 20 | 21 | 0.015 | (0.04,0.73) |
| MPrG.R | 0.66 | 5.03 | 20 | 21 | 0.0002 | (0.34,0.84) |
| MSFG.R | 0.73 | 6.62 | 20 | 21 | 3.2*10^-5^ | (0.46,0.88) |
| MTG.R | 0.70 | 5.72 | 20 | 21 | 0.0001 | (0.40,0.86) |
| OCP.R | 0.35 | 2.08 | 20 | 21 | 0.05 | (-0.07,0.67) |
| OFuG.R | 0.33 | 2.01 | 20 | 21 | 0.052 | (-0.09,0.66) |
| OpIFG.R | 0.79 | 8.82 | 20 | 21 | 3.02*10^-6^ | (0.56,0.91) |
| OrIFG.R | 0.73 | 6.62 | 20 | 21 | 3.20*10^-5^ | (0.46,0.88) |
| PCgG.R | 0.64 | 4.62 | 20 | 21 | 0.0004 | (0.31,0.83) |
| PCu.R | 0.72 | 6.38 | 20 | 21 | 4.28*10^-5^ | (0.44,0.87) |
| PHG.R | 0.42 | 2.46 | 20 | 21 | 0.023 | (0.007,0.71) |
| PIns.R | 0.39 | 2.28 | 20 | 21 | 0.03 | (-0.02,0.69) |
| PO.R | 0.36 | 2.13 | 20 | 21 | 0.04 | (-0.06,0.67) |
| PoG.R | 0.68 | 5.43 | 20 | 21 | 0.0001 | (0.38,0.86) |
| POrG.R | 0.74 | 6.94 | 20 | 21 | 2.21*10^-5^ | (0.48,0.88) |
| PP.R | 0.54 | 3.37 | 20 | 21 | 0.003 | (0.16,0.78) |
| PrG.R | 0.66 | 4.99 | 20 | 21 | 0.0002 | (0.34,0.84) |
| PT.R | 0.27 | 1.75 | 20 | 21 | 0.10 | (-0.16,0.62) |
| SCA.R | 0.40 | 2.33 | 20 | 21 | 0.029 | (-0.01,0.70) |
| SFG.R | 0.75 | 7.14 | 20 | 21 | 1.76*10^-5^ | (0.49,0.89) |
| SMC.R | 0.44 | 2.57 | 20 | 21 | 0.018 | (0.02,0.72) |
| SMG.R | 0.68 | 5.27 | 20 | 21 | 0.0001 | (0.37,0.85) |
| SOG.R | 0.23 | 1.61 | 20 | 21 | 0.142 | (-0.2,0.59) |
| SPL.R | 0.66 | 5.01 | 20 | 21 | 0.0002 | (0.34,0.84) |
| STG.R | 0.81 | 9.71 | 20 | 21 | 1.31*10^-6^ | (0.60,0.91) |
| TMP.R | 0.67 | 5.07 | 20 | 21 | 0.00024 | (0.35,0.85) |
| TrIFG.R | 0.39 | 2.29 | 20 | 21 | 0.032 | (-0.02,0.69) |
| TTG.R | 0.30 | 1.85 | 20 | 21 | 0.083 | (-0.13,0.63) |
| Accum.L | 0.06 | 1.14 | 20 | 21 | 0.37 | (-0.35,0.47) |
| Amyg.L | 0.37 | 2.18 | 20 | 21 | 0.041 | (-0.05,0.68) |
| Cau.L | 0.78 | 8.19 | 20 | 21 | 5.62*10^-6^ | (0.54,0.90) |
| Hip.L.L | 0.59 | 3.92 | 20 | 21 | 0.001 | (0.23,0.81) |
| Pal.L | 0.37 | 2.18 | 20 | 21 | 0.041 | (-0.05,0.68) |
| Put.L | 0.59 | 3.93 | 20 | 21 | 0.001 | (0.238,0.81) |
| Thal.L | 0.52 | 3.24 | 20 | 21 | 0.0049 | (0.144,0.77) |
| VentDC.L | 0.34 | 2.04 | 20 | 21 | 0.055 | (-0.08,0.66) |
| ACgG.L | 0.65 | 4.73 | 20 | 21 | 0.0004 | (0.322,0.84) |
| AIns.L | 0.40 | 2.36 | 20 | 21 | 0.028 | (-0.01,0.70) |
| AOrG.L | 0.18 | 1.44 | 20 | 21 | 0.20 | (-0.25,0.55) |
| AnG.L | 0.65 | 4.86 | 20 | 21 | 0.0003 | (0.335,0.84) |
| Calc.L | 0.44 | 2.63 | 20 | 21 | 0.016 | (0.041,0.73) |
| CO.L | 0.42 | 2.45 | 20 | 21 | 0.023 | (0.006,0.71) |
| Cun.L | 0.54 | 3.37 | 20 | 21 | 0.0039 | (0.163,0.78) |
| Ent.L | 0.47 | 2.78 | 20 | 21 | 0.012 | (0.068,0.74) |
| FO.L | 0.38 | 2.25 | 20 | 21 | 0.035 | (-0.03,0.69) |
| FRP.L | 0.58 | 3.85 | 20 | 21 | 0.001 | (0.227,0.80) |
| FuG.L | 0.62 | 4.26 | 20 | 21 | 0.0008 | (0.275,0.82) |
| GRe.L | -0.36 | 0.52 | 20 | 21 | 0.92 | (-0.64,0.12) |
| IOG.L | 0.69 | 5.55 | 20 | 21 | 0.0001 | (0.392,0.86) |
| ITG.L | 0.66 | 5.03 | 20 | 21 | 0.0002 | (0.349,0.84) |
| LiG.L | 0.76 | 7.43 | 20 | 21 | 1.26*10^-5^ | (0.508,0.89) |
| LOrG.L | 0.21 | 1.54 | 20 | 21 | 0.16 | (-0.22,0.58) |
| MCgG.L | 0.59 | 3.88 | 20 | 21 | 0.001 | (0.231,0.80) |
| MFC.L | 0.44 | 2.62 | 20 | 21 | 0.01 | (0.038,0.73) |
| MFG.L | 0.78 | 8.18 | 20 | 21 | 5.7*10^-6^ | (0.542,0.90) |
| MOG.L | 0.38 | 2.25 | 20 | 21 | 0.035 | (-0.03,0.69) |
| MOrG.L | 0.65 | 4.78 | 20 | 21 | 0.0003 | (0.327,0.84) |
| MPoG.L | 0.17 | 1.41 | 20 | 21 | 0.21 | (-0.26,0.55) |
| MPrG.L | 0.70 | 5.84 | 20 | 21 | 8.5*10^-5^ | (0.413,0.86) |
| MSFG.L | 0.37 | 2.18 | 20 | 21 | 0.041 | (-0.05,0.68) |
| MTG.L | 0.81 | 9.88 | 20 | 21 | 1.13*10^-6^ | (0.606,0.92) |
| OCP.L | 0.72 | 6.37 | 20 | 21 | 4.3*10^-5^ | (0.448,0.87) |
| OFuG.L | 0.54 | 3.38 | 20 | 21 | 0.003 | (0.164,0.78) |
| OpIFG.L | 0.53 | 3.26 | 20 | 21 | 0.004 | (0.148,0.77) |
| OrIFG.L | 0.30 | 1.89 | 20 | 21 | 0.07 | (-0.12,0.64) |
| PCgG.L | 0.52 | 3.23 | 20 | 21 | 0.005 | (0.143,0.77) |
| PCu.L | 0.64 | 4.65 | 20 | 21 | 0.0004 | (0.315,0.83) |
| PHG.L | 0.36 | 2.15 | 20 | 21 | 0.043 | (-0.05,0.68) |
| PIns.L | 0.36 | 2.13 | 20 | 21 | 0.045 | (-0.06,0.67) |
| PO.L | 0.32 | 1.95 | 20 | 21 | 0.067 | (-0.10,0.65) |
| PoG.L | 0.70 | 5.74 | 20 | 21 | 9.7*10^-5^ | (0.406,0.86) |
| POrG.L | 0.74 | 6.82 | 20 | 21 | 2.5*10^-5^ | (0.475,0.88) |
| PP.L | 0.52 | 3.16 | 20 | 21 | 0.005 | (0.132,0.77) |
| PrG.L | 0.74 | 6.91 | 20 | 21 | 2.29*10^-5^ | (0.480,0.88) |
| PT.L | 0.51 | 3.08 | 20 | 21 | 0.006 | (0.120,0.76) |
| SCA.L | 0.48 | 2.89 | 20 | 21 | 0.009 | (0.088,0.75) |
| SFG.L | 0.76 | 7.38 | 20 | 21 | 1.34*10^-5^ | (0.505,0.89) |
| SMC.L | 0.52 | 3.24 | 20 | 21 | 0.005 | (0.144,0.77) |
| SMG.L | 0.60 | 4.11 | 20 | 21 | 0.001 | (0.258,0.81) |
| SOG.L | 0.48 | 2.89 | 20 | 21 | 0.009 | (0.088,0.75) |
| SPL.L | 0.71 | 5.94 | 20 | 21 | 7.5*10^-5^ | (0.420,0.87) |
| STG.L | 0.62 | 4.37 | 20 | 21 | 0.0007 | (0.286,0.82) |
| TMP.L | 0.90 | 20.0 | 20 | 21 | 1.68*10^-9^ | (0.784,0.96) |
| TrIFG.L | 0.62 | 4.35 | 20 | 21 | 0.0007 | (0.284,0.82) |
| TTG.L | 0.01 | 1.02 | 20 | 21 | 0.47 | (-0.40,0.42) |
